# Metagenomic insights into microbial communities of terrestrial mud volcanos: functional diversity of subsurface archaea and bacteria

**DOI:** 10.3389/fmicb.2026.1892847

**Published:** 2026-08-11

**Authors:** Vitaly V. Kadnikov, Andrey V. Mardanov, Alexey V. Beletsky, Nikolai V. Ravin

**Affiliations:** Institute of Bioengineering, Research Center of Biotechnology of the Russian Academy of Sciences, Moscow, Russia

**Keywords:** ANME, candidate phylum EX4484-52, deep subsurface, metagenome-assembled genome, terrestrial mud volcano

## Abstract

Terrestrial mud volcanoes are surface geological features where fluidized sediments and gasses from the subsurface are discharged along a fracture network providing a window into the deep biosphere. Although mud volcanoes constitute an important source of methane emission from natural environments, their microbial communities responsible for methane cycling remain poorly characterized. Using a metagenomics approach, we investigated the taxonomic composition and metabolic potential of microbial communities in three active mud volcanoes in the Kerch-Taman mud volcanic province. Despite the volcanoes’ close proximity their microbial communities strongly differ. In the Kmv1 and Kmv2 volcanoes surface horizons mostly harbored organotrophic microbial communities, while the relative abundance of anaerobic methanotrophic archaea (ANME) increased with depth. The deep horizons (1.5 m) of Kmv1 were dominated by *Ca.* Methanoperedenaceae that lacked nitrate reductase and could couple methane oxidation to the reduction of metal oxides, while the abundance of sulfate-reducing bacteria was low. Consistently, with higher sulfate content, the deep horizon in Kmv2 was dominated by *Ca.* Methanoperedenaceae, ANME-2a/2b clade, sulfate-reducing *Desulfobacterota* and sulfur-oxidizing *Gammaproteobacteria*. No clear depth distribution of taxa was observed in the Kmv3 volcano where microorganisms of the methane and sulfur cycles, namely, methanogens, ANME-3 clade, methanotrophic bacteria, and sulfate reducers were simultaneously detected. A high-quality genome of a member of the archaeal candidate phylum EX4484-52 within the DPANN lineage was assembled from metagenomes. This archaeon, named *Candidatus Lutivulcanarchaeum fermentans*, has complete glycolytic pathway and ATP generation mechanisms, but lacked the biosynthetic pathways for many key cellular compounds, indicating a parasitic or symbiotic lifestyle.

## Introduction

The Earth’s deep subsurface biosphere—from marine sediments and continental subsurface to groundwater aquifers and hydrothermal systems—is considered one of the planet’s largest reservoirs of microbial diversity and biomass. Current estimates indicate that a significant part of prokaryotic biomass is concentrated in subsurface environments, where microorganisms thrive under extremely low energy fluxes and limited organic carbon availability (Magnabosco et al., 2018). Such ecosystems are characterized by pronounced stratification of physicochemical parameters, including gradients of pressure, temperature, mineralization, dissolved gas concentrations, and the distribution of electron donors and acceptors. Geochemical heterogeneity creates a mosaic of ecological niches in which microorganisms adapt to conditions of energy deficiency, high salinity, and low oxygen conditions. Recent metagenomic studies highlight that redox gradients determine the spatial organization of microbial communities and their metabolic networks in deep sediments and subsurface reservoirs (Schauberger et al., 2024).

Of particular interest are mud volcanoes, associated with active migration of gas-fluid flows from deep hydrocarbon-bearing sedimentary basins to the surface through a fracture network that often extends several kilometers in depth (Mazzini and Etiope, 2017). Mud volcanoes are typically cone-shaped circular structures or pools from which gaseous mud slurries are discharged (Dimitrov, 2002; Kopf, 2002). Mud volcanos are unique natural structures that bring deep fluids, finely dispersed clay material, and gasses, often enriched in methane and hydrogen sulfide, to the surface. The fluids and gasses contain various organic and inorganic compounds that can be used by various metabolic groups of microorganisms. These systems are considered natural windows into the deep subsurface biosphere, allowing the study of microbial communities that form under conditions inaccessible to direct drilling or long-term monitoring. Mud volcanoes can play a significant role in regional and global methane balance, serving both as sources and as sites for its microbial transformation (Mazzini and Etiope, 2017). Recent studies have confirmed that in mud volcanic systems, deep methane processing is associated with the activity of both aerobic methanotrophs and anaerobic methanotrophic archaea (ANME), and the intensity of microbial methane transformation is mostly determined by geochemical gradients of sulfate and iron in ascending fluids (Slobodkin et al., 2024; Chen et al., 2024; Rajendran et al., 2025).

Microbial communities of submarine and terrestrial mud volcanoes differ, in part because the main electron acceptor in submarine mud volcanoes is sulfate, while in terrestrial mud volcanoes it is likely iron minerals (Tu et al., 2017). Historically, most research on microbial communities associated with mud volcanoes has focused on those located on the seafloor (Martinez et al., 2006; Niemann et al., 2006; Lösekann et al., 2007; Klasek et al., 2019; Collado et al., 2026). In recent years, research into terrestrial mud volcanoes has expanded rapidly. These studies revealed that archaeal communities are mostly composed of methanogens and ANME archaea, while bacterial communities are more diverse both phylogenetically and metabolically, and consist of members of various phyla capable of carrying out diverse processes such as sulfate reduction, oxidation of reduced sulfur compounds, aerobic oxidation of methane, destruction of organic compounds through fermentation, aerobic and anaerobic respiration, and others (Cheng et al., 2012; Wrede et al., 2012; Lin et al., 2018; Ren et al., 2018; Tu et al., 2017; Wang et al., 2014; Nagarajan et al., 2023; Tu et al., 2022; Megyes et al., 2021; Miyake et al., 2023; Slobodkin et al., 2024; Chen et al., 2024). Overall, the available data point to active methane, sulfur, nitrogen, and iron cycling.

The Kerch-Taman mud volcanic province, located in the Kerch peninsula (the south-eastern end of Crimea) and the Taman peninsula between the Sea of Azov and the Black Sea is one of the most manifested mud volcanism areas in Eurasia hosting more than 100 active mud volcanoes (Kopf, 2002). The total thickness of the sedimentary strata in this region reaches 10 km, and the hydrochemical data suggest that fluids discharging through mud volcanos in this region are formed at depths ranging from 1 to 4.5 km (Lavrushin et al., 2003). The Kerch-Taman region is characterized by a high number of mud volcanoes of various morphological and hydrodynamic types, from periodically active to those with a continuous liquid fluid and gas emissions (Lavrushin et al., 2021). The region’s geological activity results in the formation of complex fluid systems with variable salinity and redox conditions. This variability makes these features a model platform for analyzing microbial communities under subsurface fluid discharge conditions and allows for comparison of the taxonomic structure of communities with geochemical environmental parameters.

Although several new taxa of bacteria were isolated from Taman mud volcanoes (e.g., Khomyakova et al., 2022; Khomyakova et al., 2023; Slobodkina et al., 2022; Slobodkina et al., 2023), only a few studies analyzed these communities using 16S rRNA gene profiling and metagenomics methods. The first study analyzed the microbial community of bubbling fluids retrieved from an active mud volcano in in the Kerch peninsula using a metagenomics approach (Mardanov et al., 2020). The microbial community was dominated by chemolithoautotrophic sulfur-oxidizing *Campylobacterota* and *Gammaproteobacteria*, aerobic *Methylococcales* bacteria and ANME archaea of the *Ca.* Methanoperedenaceae family (ANME-2d). The later lacked nitrate reductase and probably couple methane oxidation to the reduction of metal oxides (Mardanov et al., 2020). Two other metagenomic studies were focused on the eastern part of the Kerch-Taman region, the Taman Peninsula. Merkel et al. (2021) founded that microbial community of one of the examined volcanoes was dominated by methane-oxidizing archaea belonging to ANME-3 group (Merkel et al., 2021). The subsequent study investigated the taxonomic composition and metabolic potential of microbial communities in five mud volcanoes (Slobodkin et al., 2024). Aerobic and anaerobic methanotrophs, sulfur or iron reducers, and facultative anaerobes with broad metabolic capabilities, were detected. Notably, the most numerous group of anaerobic methanotrophs was ANME-2a/2b (*Ca.* Methanocomedenaceae), although in some samples other ANME lineages were also found.

The main objective of this study was to uncover the composition and functional potential of microbial communities involved in methane and sulfur cycling in mud volcanoes located on the western part of the Kerch-Taman region, the Kerch Peninsula. In this paper, we report the taxonomic composition of microbial communities of nine fluid samples obtained from three mud volcanoes at different depths, as well as metagenomic analysis of three deepest samples most relevant to indigenous subsurface biosphere. We obtained 88 high-quality metagenome-assembled genomes (MAGs) and assessed metabolic capabilities of the dominant community members, enabling metabolic reconstruction of the microbial community, and revealing the primary microbial drivers of the methane, sulfur and nitrogen cycles.

## Materials and methods

### Characterization of sampling sites, chemical analysis and DNA extraction

**Figure 1:**
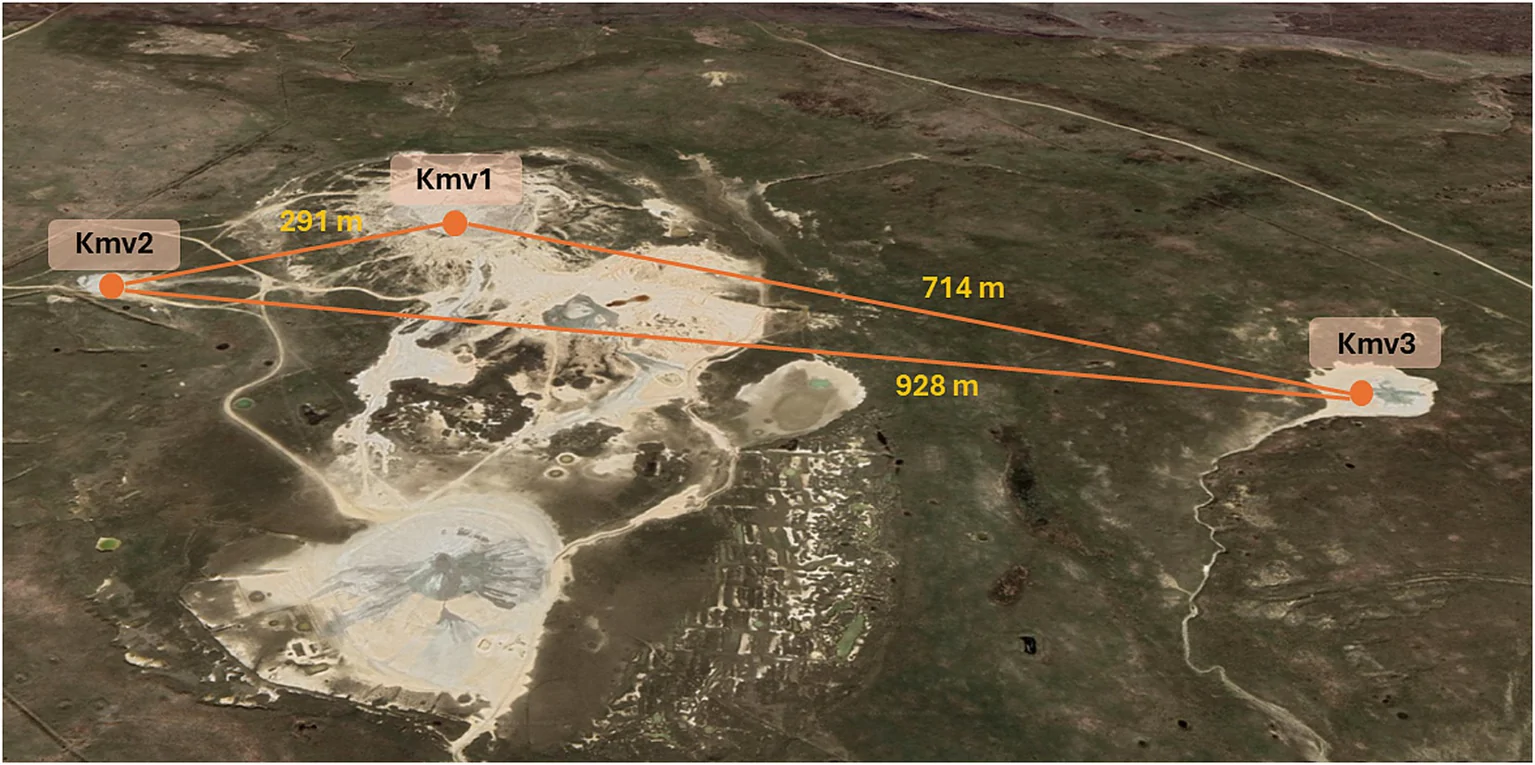
Mud volcanoes explored. Kmv1, Andrusov; Kmv2, Tishchenko; Kmv3, Oldenburg. The image was reproduced from Google Earth (accessed 07 October 2025). Imagery © 2025 Google, Airbus.

The studied mud volcanoes are located on the Bulganak mud volcano field near the village of Bondarenkovo, in the vicinity of the city of Kerch (Figure 1). Mud fluid samples were collected on September 23, 2022, from three volcanoes: Andrusov (Kmv1), Tishchenko (Kmv2) and Oldenburg (Kmv3). Fluid samples from each volcano were collected from three different depths in sterile 50 mL test tubes, avoiding the capture of gas bubbles (Table 1). Sampling was carried out using a syringe, which was lowered to the desired depth. The fluid was drawn into a syringe by pulling up a piston tied to a rope. Temperature, pH and redox potential (Eh) of the fluids collected at a depth of 20 cm were determined directly at the sampling site using an HI 8314 pH meter (Hanna Instruments, Germany).

**Table 1:** Fluid samples analyzed by molecular methods.

Sample ID	Depth	Volcano name	Coordinates
Kmv1_20	20 cm	Andrusov	45.427217, 36.477114
Kmv1_70	70 cm		
Kmv1_150	150 cm		
Kmv2_20	20 cm	Tishchenko	45.426114, 36.473695
Kmv2_70	70 cm		
Kmv2_150	150 cm		
Kmv3_20	20 cm	Oldenburg	45.424696, 36.485551
Kmv3_100	100 cm		
Kmv3_180	180 cm		

For chemical analysis, fluid samples collected from a depth of 20 cm were centrifuged at 12,000 ×*g* for 10 min. The resulting supernatant was filtered through sterile membrane filters with a pore size of 0.22 μm (Merck Millipore, Darmstadt, Germany) and then analyzed by inductively coupled plasma mass spectrometry (ICP-MS) and ion chromatography.

The gas content in the mud samples was determined by phase equilibrium degassing followed by headspace gas chromatography using a Kristall-2000-M chromatograph (Chromatec, Russia) equipped with a flame ionization detector. After equilibration, an aliquot of the headspace gas was analyzed, and the contents of gasses were expressed as volume percentages of the total analyzed headspace gas phase.

### DNA extraction, 16S rRNA gene library preparation and sequencing

Total DNA from 15 mL of mud slurry was isolated using the DNeasy PowerMax Soil Kit (Qiagen, Hilden, Germany) according to the manufacturer’s instructions. The DNA yield was approximately 100 ng per sample. Amplification of the 16S rRNA gene fragments covering the V3–V4 variable regions was performed using universal prokaryotic primers 341F_Fr (5′-CCT AYG GGD BGC WSC AG-3′) and 806R_Fr (5′-GGA CTA CNV GGG THT CTA AT-3′). The resulting PCR products were indexed using the Nextera XT Index Kit v2 (Illumina, San Diego, CA, USA). After indexing, the amplicons were combined into a single library and sequenced on the Illumina MiSeq platform in paired-end mode (2 × 300 bp).

Paired overlapping reads were merged using FLASH v.1.2.11. The resulting dataset included 967,226 16S rRNA gene sequences. The resulting sequences were grouped into operational taxonomic units (OTUs) at a 97% similarity threshold using USEARCH v.11 (Edgar, 2010). Taxonomic identification of OTUs was performed by comparison with the SILVA v.138.1 database using the VSEARCH v. 2.28.1 algorithm (Rognes et al., 2016). To estimate the relative abundance of OTUs, all reads from each sample were mapped to OTU sequences at a global identity threshold of 97% using USEARCH. Alpha diversity indices were calculated at the 97% OTU level using USEARCH v.11. The data were rarefied to account for sequencing depth bias.

### Analysis of beta-diversity

The beta diversity of microbial communities from mud volcanoes worldwide was estimated based on 16S rRNA gene data using a weighted UniFrac distance matrix. Reads were aligned using Mothur v.1.47.0 (Schloss et al., 2009) against the Silva seed v.138.2 database. Because different 16S rRNA gene fragments were sequenced for different samples, coordinates 355–633 in the alignment were identified, which intersect the majority of reads (>90%) from all samples. Only reads within these coordinates were used for further analysis. Clustering of reads into OTUs and calculation of UniFrac distances between samples based on the OTU table were performed using USEARCH v.11. Pairwise UniFrac distances were visualized as a heatmap using the heatmap.2 function in R v.4.5.2. Samples were hierarchically clustered using the complete linkage method.

Beta diversity analysis for the nine fluid samples analyzed in this study was performed similarly, but using the obtained sequences of the V3–V4 region of the 16S rRNA gene. Sample similarity visualization using the UniFrac metric using principal coordinate analysis (PCoA) was performed using the ggplot2 library and the cmdscale function in R.

### Metagenome sequencing and MAGs assembly

Metagenomic analysis was performed on mud fluid samples from three volcanoes collected at the greatest depths. Metagenomic DNA samples were sequenced on the Illumina platform (Illumina, San Diego, CA, USA). Libraries prepared using the NEBNext Ultra II DNA Library Prep Kit were sequenced on an Illumina HiSeq2500 in paired-end sequencing mode (2 × 150 bp). For Kmv1_150, Kmv2_150, and Kmv3_180 samples, 107, 121, and 95 million read pairs, respectively, were obtained, corresponding to approximately 32, 36, and 28 Gb of nucleotide sequences.

Adapters were removed using Cutadapt v.4.8 (Martin, 2011), and low-quality regions at the ends of reads were removed using Sickle v.1.33. Contig assembly was performed using SPAdes v.4.0.0 (Nurk et al., 2017). Assembled contigs were binned in using five programs: COMEBin v.1.0.4 (Wang et al., 2024), Semibin2 v.1.5.1 (Pan et al., 2023), MetaBAT2 v.2.15 (Kang et al., 2019), Concoct v.1.1.0 (Alneberg et al., 2014), and MaxBin2 v.2.2.7 (Wu et al., 2016). The binning results were integrated into an optimized set of genomic bins using DAS Tool v.1.1.7 (Sieber et al., 2018). MAG completeness and the level of redundancy (potential contamination) were assessed using CheckM2 v.1.0.2 (Chklovski et al., 2023). The taxonomic affiliation of the reconstructed MAGs was determined using GTDB-Tk v.2.4.1 (Chaumeil et al., 2022) and the Genome Taxonomy Database (GTDB) release 232 (Parks et al., 2018).

For each MAG, the closest reference genome in the GTDB tree was determined using the ape v.5.6–2 package in the R environment. The ANI (average nucleotide identity) and AAI (average amino acid identity) values relative to the closest reference genomes were calculated using scripts from the Enveomics Collection (Rodriguez-R and Konstantinidis, 2016).

Annotation of the genomes was performed employing the NCBI Prokaryotic Genome Annotation Pipeline (Tatusova et al., 2016) and the RAST server (Aziz et al., 2008). Functional annotation of genes against the KEGG database was performed using the KAAS web server, and the completeness of KEGG modules was assessed using the KEMET Python tool (Palù et al., 2022). Prediction and comparative analysis of the metabolic capabilities of MAGs representing the EX4484-52 lineage was performed using the DRAM tool (Shaffer et al., 2020).

### Phylogenetic analysis

120 single-copy marker genes were identified in analyzed genomes, concatenated, and aligned using the GTDB-Tk v.2.4.1 tool. PhyML v. 3.3 (Guindon et al., 2010) was used to construct the maximum likelihood phylogenetic tree. Branch support values were estimated using the Bayesian test in PhyML.

### Comparative analysis of gene abundancies in metagenomes

To compare relative gene abundancies in the metagenomes of fluid samples from three volcanoes, genes were predicted in all contigs using Prodigal v.2.6.3 (Hyatt et al., 2010) with the meta-parameter. Illumina reads were mapped to the contigs using bowtie2 v.2.5.4 (Langmead and Salzberg, 2012), the number of mapped reads to each gene was counted using featureCounts v.2.1.1 (Liao et al., 2014) with “-O” parameter, and RPKM (reads per kilobase per million mapped reads) values were calculated from read counts using RPKM function of edgeR v.4.8.2 (Robinson et al., 2010) R package. A selection of genes of interest was made from the KEGG database and found by searching for gene homologs from contigs in the KOfam hmm database using hmmscan v.3.3.1 (Aramaki et al., 2020) with filters of e-value < 1e-10 and alignment score > 0.7 of hmm threshold score.

## Results and discussion

### Geochemical characteristics of mud volcano fluids

The chemical composition of fluids reflects the complex interaction of marine pore waters, deep fluid flows, and ascending gasses. Such systems are formed by fluid migration along faults and dehydration of sedimentary strata, leading to the mixing of various geochemical reservoirs. According to current concepts, the hydrogeochemical regime of mud volcanoes is controlled by a combination of the lithological composition of sediments, the depth of the reservoirs, and the intensity of fluid discharge (Judd, 2005; Niemann, 2020).

Negative Eh values (Table 2) indicate reduced environmental conditions. These values are consistent with active microbial sulfate reduction, as well as possible chemical reactions reducing sulfur and iron compounds in the fluids. Such redox potential values are also characteristic of subsurface marine sediments, where oxygen and nitrate are rapidly consumed resulting in stable anaerobic conditions (Orcutt et al., 2013).

**Table 2:** Physicochemical characteristics of the aqueous phase of the mud fluid.

Parameter (unit)	Kmv1*	Kmv2*	Kmv3*
Temperature (C)	17.6	16.1	17.1
pH	7.54	7.80	7.61
eH (mV)	−178	−254	−240
Cl¯ (mg/L)	3598.7	3472.7	3196.4
NO_3_¯ (mg/L)	13.1	13.9	11.4
PO_4_^3−^ (mg/L)	7.6	3.5	0
SO_4_^2−^(mg/L)	7.8	56.8	4
Li (mg/L)	2.44	1.62	2.27
Na (mg/L)	3,875	4,164	3,672
Mg (mg/L)	20.35	63.19	28.07
Al (mg/L)	0.065	0.045	0.049
Si (mg/L)	16.84	13.29	27.11
P (mg/L)	3.9	1.95	0.9
К (mg/L)	27.17	40.94	53.22
Ca (mg/L)	64.13	81.16	79.04
Fe (mg/L)	0.59	0.61	0.48
Sr (mg/L)	2.43	2.32	10.76
Ba (mg/L)	1.23	1.34	2.65

*Samples collected at a depth of 20 cm.

The sampled fluids were characterized by near neutral pH, elevated mineralization and a predominance of sodium chloride salinity with relatively low sulfate concentrations and moderate calcium and magnesium contents (Table 2).

The sodium chloride type of salinity indicates a significant contribution of the marine component to the formation of the fluid system. This ionic composition is typical of systems where seawater recirculation and subsequent modification during interaction with sedimentary rocks play a significant role. High chloride and sodium concentrations can be explained by seawater infiltration into groundwater (Chen et al., 2023) or the release of ions from clay minerals during diagenetic transformations of sediments at depth (Lazar et al., 2022; Farhadian Babadi et al., 2021). Low sulfate concentrations detected in the samples indicate long-term reducing conditions and active microbial sulfate reduction in the deep horizons of the sedimentary strata. Recent research shows that in such systems, sulfate can be almost entirely consumed by microbial consortia, resulting in the formation of reducing fluids enriched in sulfide and dissolved metals (Tu et al., 2017).

Differences in Mg, Ca, and Si concentrations in fluid samples from different volcanoes likely reflect intense localized water-rock interactions within the sedimentary strata. The elevated dissolved silicon content in Kmv3 may be related to the dissolution of aluminosilicate minerals in clay sediments and, possibly, to diagenetic transformations such as illitization and the smectite-to-illite transition. This sample also contained the highest strontium and barium concentrations, exceeding the values for Kmv1 and Kmv2 (Table 2). The high Sr^2+^ and Ba^2+^ contents in sample Kmv3 are likely due to a combination of dissolution of carbonate and sulfate minerals such as celestite (SrSO_4_) and barite (BaSO_4_) and remineralization processes of organic matter in the microenvironments formed during its decomposition (Steiner et al., 2025).

Variations in Ca^2+^ and Mg^2+^ concentrations, in turn, may indicate ion exchange processes on the surface of clay minerals, as well as a shift in the carbonate equilibrium (precipitation/dissolution of calcite and dolomite) with changes in pH and partial pressure of CO₂. Thus, the chemical composition of the fluids is formed both by the inherited marine component and as a result of secondary diagenetic transformations (Huang et al., 2015; Nikitenko and Ershov, 2021; Ijiri et al., 2023). It should be noted that although sulfate concentrations in the fluids were low, they varied greatly between the studied volcanoes, which likely reflects local features of the chemical composition of sedimentary rocks and/or the activity of microbial processes in the sulfur cycle.

An additional geochemical feature is the presence of dissolved redox-active metals, primarily iron whose concentrations reach ~0.01 mM, which may indicate reductive dissolution of iron-containing minerals (Fe(III) oxyhydroxides, clay phases, sulfides) in an anaerobic environment. Such concentrations are characteristic of zones of active redox stratification, where iron is involved in microbial-mediated cycles. Recent studies show that Fe(III) can serve as a terminal electron acceptor in dissimilatory iron reduction processes, while Fe(II) in the presence of oxidizing agents (O₂, NO₃^−^) can participate in reverse reactions, forming a closed iron cycle (Sokol et al., 2018; Kappler et al., 2021). In mud volcano and cold seep ecosystems, such processes create additional electron transport pathways and can compete with methanogenesis and sulfate reduction for organic substrate and hydrogen. Thus, iron not only reflects the redox state of the environment but also potentially controls the direction and intensity of microbial metabolic pathways.

Comparative analysis with mud volcanoes in other regions reveals both general patterns and regional differences. In Azerbaijan and the Caspian region, fluids are also chloride-sodium rich and sulfate-deficient, but are often characterized by higher temperatures and higher feeder depths, which is associated with petroleum-bearing structures and the contribution of thermogenic hydrocarbons (Alizadeh et al., 2016). Under these conditions, the influence of deep fluids and thermocatalytic processes is more pronounced. In contrast, mud volcanoes in Romania (Berca-Arbanesh) are characterized by lower mineralization (5–15‰) and more oxidizing conditions, which was interpreted as a result of shallower circulation and intense mixing with meteoric and surface waters (Brustur et al., 2015).

The gas phase of samples Kmv1–Kmv3 (Table 3) is characterized by the dominance of molecular nitrogen and oxygen (N₂ 75–79%; O₂ 5–7%) with a low content of hydrocarbon components (CH₄ 0.21–0.66%; C₂/C₁ ≤ 0.0022). This composition indicates a high degree of atmospheric mixing in the near-surface degassing zone and an initially low gas content of the ascending fluids. Moreover, the low values of the C₂/C₁ (ethane/methane) ratio correspond to the range typical of microbial (biogenic) methane formed as a result of anaerobic methanogenesis in the sedimentary strata. As is known, thermogenic methane is characterized by higher concentrations of heavy hydrocarbons and, accordingly, increased C₂/C₁ values, while microbial systems are distinguished by almost “dry” methane with minimal admixtures of C₂^+^ components (Cesar et al., 2021; Moore et al., 2018).

**Table 3:** Composition of gas in mud volcano fluids.

Gas content (% of the total volume)	Kmv1*	Kmv2*	Kmv3*
N_2_	79.48	75.88	79.11
O_2_	5.333	7.3	5.98
H_2_	0.002	0	0.005
CO_2_	0.053	0.039	0.036
CH_4_	0.45	0.21	0.66
Ethane	0.001	0	0
Propane	0.054	0	0.002
Isobutane	0.043	0	0

*Samples collected at a depth of 20 cm.

Comparable geochemical parameters have been recorded in mud volcanoes of Azerbaijan and the Taman Peninsula, where methane is predominantly formed as a result of microbial degradation of organic matter under reducing conditions (Shnyukov and Yanko-Hombach, 2020; Lavrushin et al., 2003, 2015). Modern isotope studies (δ^13^C-CH₄, δD-CH₄) show that microbial methane in such systems can be generated at relatively low temperatures and at shallow depths, especially in zones of active fluid circulation and increased organic substrate (Mazzini and Etiope, 2017). Thus, the combination of molecular indicators (low C₂^+^ content, extremely low C₂/C₁) and regional analogies supports the dominance of microbial methanogenesis.

Thus, the fluids of Kerch mud volcanoes combine features characteristic of offshore marine systems, − high mineralization, reducing conditions, and the presence of biogenic methane, with regional characteristics, including relatively low temperatures and elevated dissolved metal contents.

Interestingly, despite the close proximity of the three volcanoes (within 1,000 m), the chemical composition of the fluids differs in some characteristics, reflecting the properties of the host rocks and potentially influencing the composition of microbial communities.

### Microbial communities structures revealed by 16S rRNA gene profiling

High-throughput sequencing of 16S rRNA gene amplicons (V3-V4 regions) allowed us to characterize the phylogenetic structure of nine microbial communities sampled from three mud volcanoes at three depths each. The analysis yielded between 38,078 and 67,029 16S rRNA gene fragment sequences per sample. It should be noted that although this approach allows for the quantitative detection of even minor phylotypes, these short 16S rRNA gene fragments are prone to PCR amplification biases and provide limited taxonomic resolution, especially at the genus/species levels, thus limiting an accurate assessment of microbial diversity compared to shotgun metagenomics. Nevertheless, because only few previous studies of terrestrial mud volcanoes have used shotgun metagenomics, we employed the 16S rRNA gene profiling for comparative analysis of all our samples and their comparison with data from other mud volcanoes obtained using the same approach.

Alpha diversity indices indicate moderately high taxonomic richness in the communities. Chao1 index values (606–2,130) indicate a significant pool of rare phylotypes, while relatively low Shannon index values (1.54–2.78) reflect the pronounced dominance of individual taxa and the uneven distribution of their relative abundances.

The presence of dominant phylotypes was most clearly revealed by specialized community structure indices (Table 4). High dominance values indicate that, despite high taxonomic richness, communities are characterized by a markedly uneven distribution of relative taxa abundances. The Jost D indices confirm this pattern: the effective number of taxa is much lower than the observed Chao1, indicating a high contribution of a limited number of dominant phylotypes to the overall community. The Jost D values highly varied between samples, reflecting pronounced differences between communities in the abundance of dominant phylotypes (Table 4).

**Table 4:** Alpha diversity indices of microbial communities of mud volcanoes at different depths.

Sample	Berger Parker	Chao1	Simpson	Shannon	Jost D	Dominance
Kmv1_20	0.14	1528.00	0.03	2.39	79.90	0.97
Kmv1_70	0.04	2125.10	0.01	2.78	273.50	0.99
Kmv1_150	0.34	606.10	0.13	1.55	12.70	0.87
Kmv2_20	0.08	1861.90	0.02	2.47	106.20	0.98
Kmv2_70	0.10	2130.90	0.02	2.58	124.20	0.98
Kmv2_150	0.12	1175.50	0.04	2.03	42.90	0.96
Kmv3_20	0.35	868.40	0.14	1.58	11.70	0.86
Kmv3_100	0.29	1118.80	0.10	1.78	17.70	0.90
Kmv3_180	0.42	928.10	0.19	1.54	9.25	0.82

Interestingly, samples collected at intermediate depths showed the highest diversity, as indicated by the Chao1 and Shannon indices. These samples likely contained OTUs characteristic of both the upper and lower horizons.

**Figure 2:**
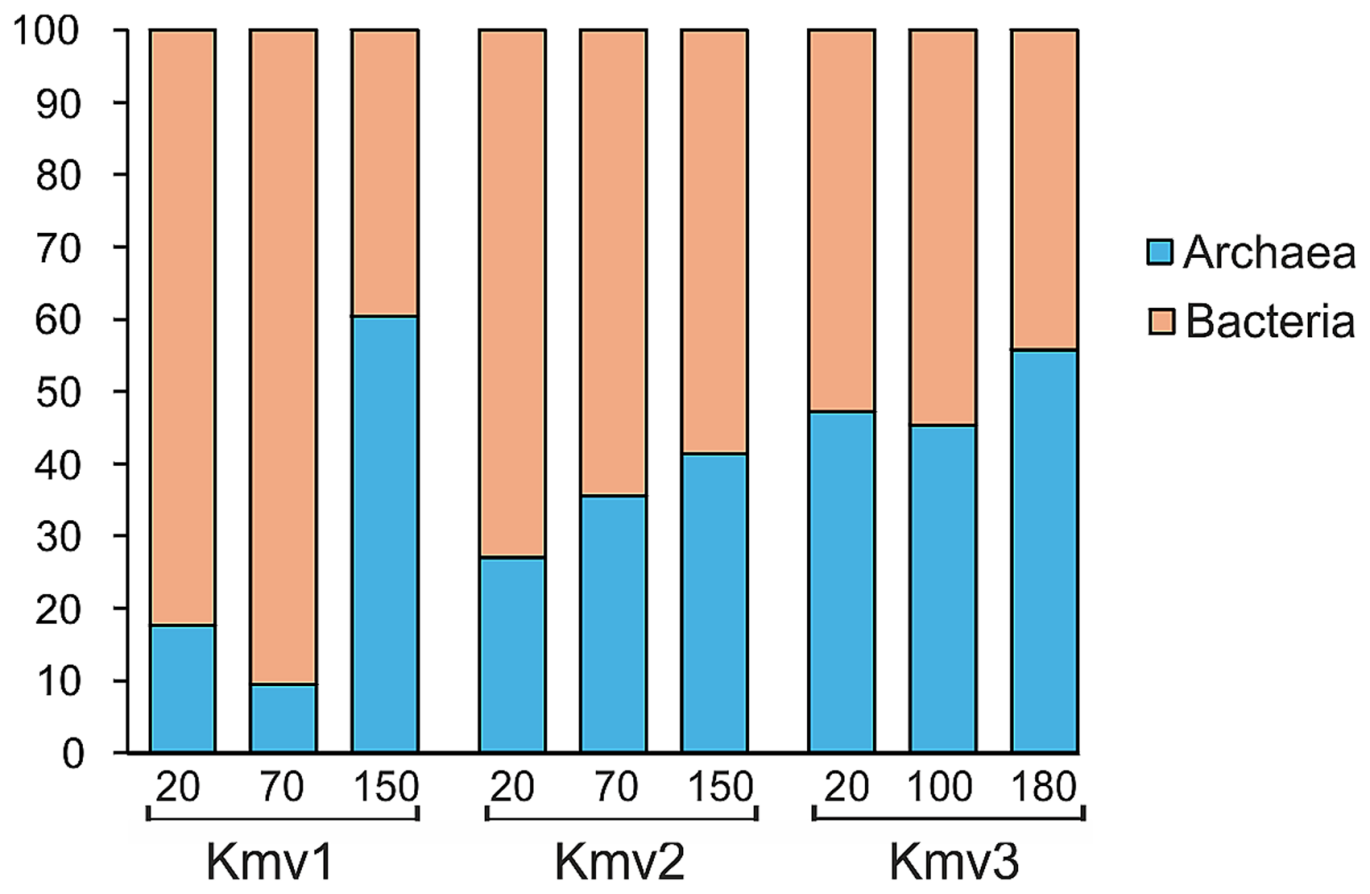
Relative abundance of bacteria and archaea in microbial communities according to 16S rRNA gene profiling data.

According to the 16S rRNA gene profiling data, archaea constituted significant fractions of the communities, and their relative abundance increased with sampling depth (Figure 2).

**Figure 3:**
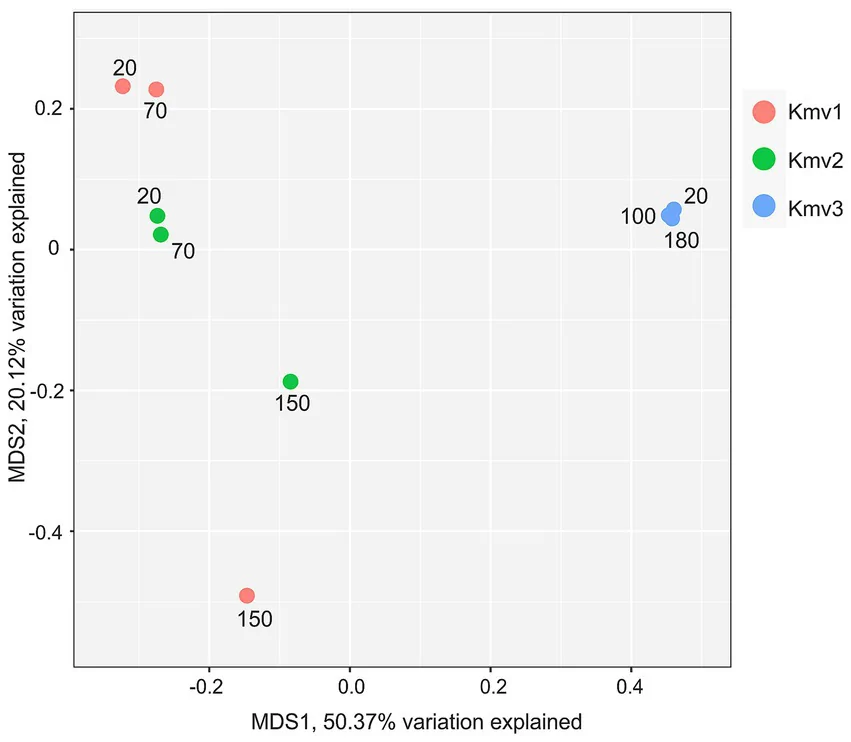
Principal coordinate analysis (PCoA) of microbial communities based on Weighted UniFrac distances calculated from 16S rRNA gene profiles. The depths from which the corresponding samples were taken are indicated next to the circles.

A principal coordinate analysis (PCoA) plot constructed using weighted UniFrac distances revealed a complex clustering pattern (Figure 3). All samples from Kmv3 formed a single, dense cluster, indicating slight variation in its microbiome composition with depth. Samples collected from depths of 20 cm and 70 cm at Kmv1 and Kmv2 also clustered by volcano, not according to sampling depth. However, samples collected from a depth of 150 cm clustered separately, indicating that the microbial communities of the deep fluid layers differed from those of the overlying horizons.

**Figure 4:**
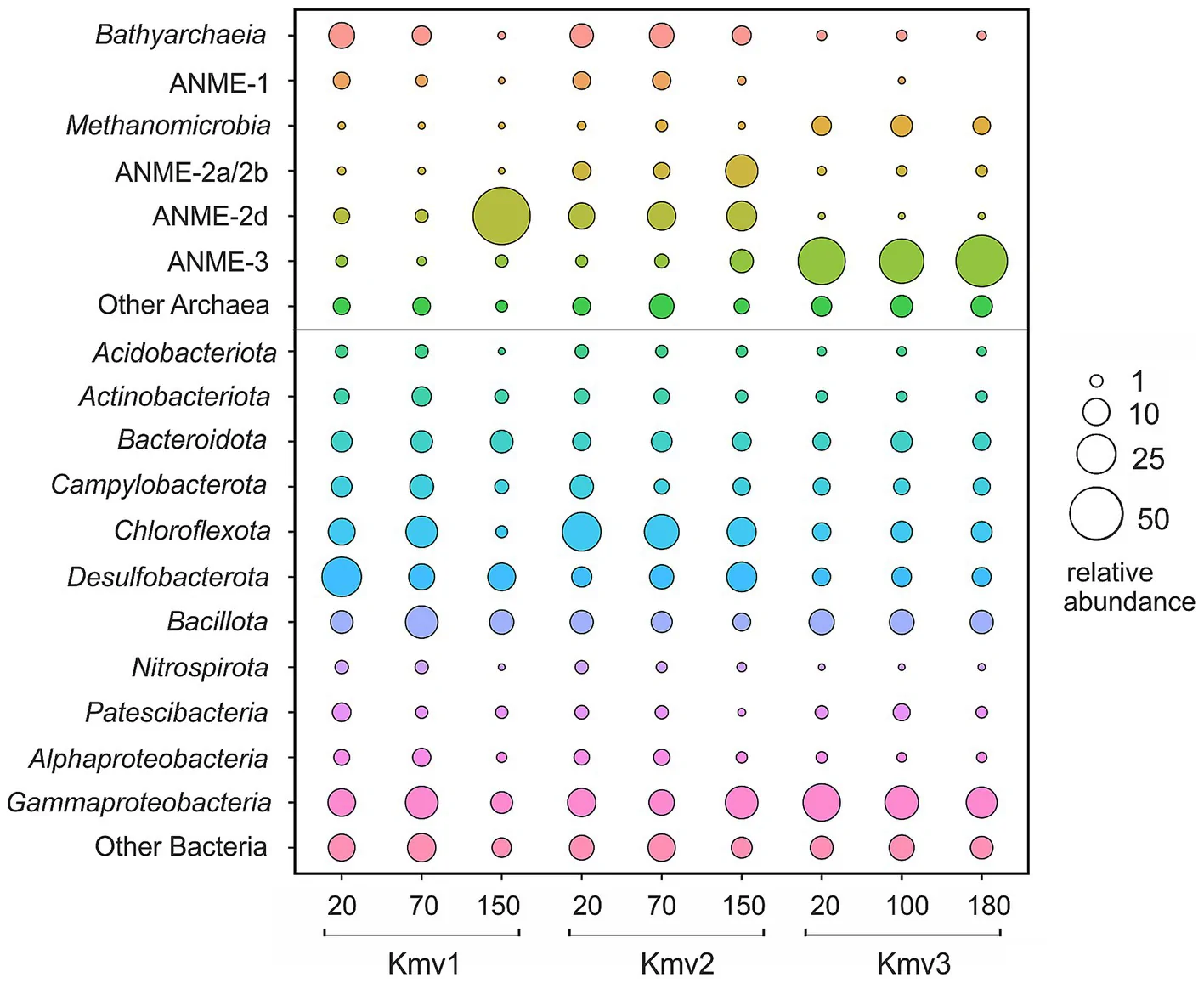
Relative abundance of taxonomic groups of microorganisms based on 16S rRNA gene profiling. Bacteria are shown at the phylum or class (for *Pseudomonadota*) level. For Archaea, the abundance of members of the classes *Bathyarchaeia* and *Methanomicrobia*, as well as the detected ANME groups, is shown.

The results of taxonomic profiling of microbial community compositions based on 16S rRNA genes are presented in Figure 4.

Archaea were predominantly represented by the phylum *Halobacteriota* (class *Methanosarcinia*), the share of which increased with depth. In the upper and middle horizons of the Kmv1 and Kmv2 volcanoes, representatives of the class *Bathyarchaeia* (phylum *Crenarchaeota*) made up significant fractions of the communities. Among the minor archaeal groups, *Thermoplasmatota* and *Nanobdellota* were identified. *Desulfobacterota, Bacillota, Pseudomonadota* (class *Gammaproteobacteria*), and *Chloroflexota* dominated among bacteria in these communities.

The distribution of archaeal ANME groups was heterogeneous: ANME-2a/2b was more abundant in Kmv2, reaching 15.5% at 1.5 m depth. ANME-1 was characteristic of the surface and middle horizons of Kmv1 and Kmv2 and was virtually absent in Kmv3. The proportion of *Ca.* Methanoperedenaceae (ANME-2d) increased with depth in Kmv1 and Kmv2, comprising 58% of the total microbial community at 1.5 m depth in Kmv1. ANME-3 dominated the archaeal ANME community in Kmv3 at all horizons. Methanogenic archaea constituted more than 1% of the communities only in the Kmv3 volcano (3–5% of the entire community at all depths) and were close to *Methanocalculus chunshungensis* (99% identity by the 16S rRNA gene sequence).

The observed depth distribution of microbial taxa abundance, clearly visible in Kmv1 and Kmv2 volcanoes, is consistent with the proposed redox stratification of fluids. Surface horizons likely correspond to zones of higher oxidation potential and intense organic matter destruction. With increasing depth, an increase in the relative abundance of *Halobacteriota* (mostly ANME lineages) was observed, indicating an increasing role of strictly anaerobic metabolic processes. A similar change in microbial assemblages along redox gradients has previously been described for methane seeps and subsurface marine sediments, where the vertical community structure reflects a sequence of aerobic, sulfate-reducing, and methanogenic zones (Knittel and Boetius, 2009; Ruff et al., 2024; Brankovits and Pohlman, 2020).

An increase in the relative abundance of ANME-2a/2b archaea in Kmv2 volcano at depth of 150 cm, accompanied by an increase in the share of *Desulfobacterota*, including sulfate-reducing lineages, may indicate the formation of an anaerobic methane oxidation zone coupled with sulfate reduction. Spatial colocalization of methanotrophic archaea and sulfate-reducing bacteria is a characteristic feature of such consortia and has been well described for marine mud volcanoes and cold seeps (Boetius et al., 2000; Orphan et al., 2002; Wegener et al., 2016). Current research shows that such microbial associations function as integrated metabolic systems, where electron transfer between partners is carried out through complex mechanisms of interspecies interaction, including direct electron transfer and the participation of multiheme cytochromes (Yu et al., 2025).

Unlike ANME-2, ANME-1 archaea were found in Kmv1 and Kmv2 volcanoes primarily at near-surface and mid-depth levels. This pattern may reflect ecological plasticity of this group and its ability to thrive under changing redox conditions. Although ANME-1 typically perform AOM in syntrophic consortia with sulfate-reducing bacteria, it was recently shown that these archaea possess ancient pathways for hydrogen metabolism and are capable of switching between oxidation and methane formation depending on environmental redox conditions (Laso-Pérez et al., 2023).

In addition to ANME archaea, aerobic methanotrophs belonging to the families *Methylococcaceae* and *Methylomonadaceae* (class *Gammaproteobacteria*, order *Methylococcales*) were detected. The abundance of *Methylococcaceae* in all samples did not exceed 0.1%, while the content of *Methylomonadaceae* reached 0.3% in Kmv1, 2% in Kmv2, and 11% in Kmv3 (Table 5). *Methylomonadaceae* are aerobic methanotrophs known as dominant active methane oxidizers in freshwater lakes (Deng et al., 2024). It should be noted that *Methylomonadaceae* can adapt to oxygen-deficient conditions and potentially use alternative electron acceptors, such as nitrate or iron (Fe^3+^), for survival (Deng et al., 2024). The presence of *Methylomonadaceae* is consistent with data from marine mud volcanoes and seeps, where aerobic methanotrophs of the order *Methylococcales* are associated with oxidized microzones of methane-saturated sediments and form the upper aerobic part of the methane filter (Niemann et al., 2006; Lösekann et al., 2007; Ruff, 2020). The dominant detected methanotroph was an organism phylogenetically close to *Methyloprofundus sedimenti*, an obligate methanotroph from oceanic sediments (Tavormina et al., 2015). The second most numerous group of aerobic methanotrophs was the genus *Methylomicrobium*. Their closest relative is *Methylomicrobium alcaliphilum*, which is consistent with literature data on the distribution of halo(alkali)philic and halotolerant aerobic methanotrophs in saline and chemically extreme habitats (Kalyuzhnaya et al., 2008).

**Table 5:** Relative abundance of *Methylomonadaceae* in mud fluid microbiomes (% of total 16S rRNA gene sequences).

Level	Kmv1	Kmv2	Kmv3
Upper	0.03	0.12	10.95
Intermediate	0.33	0.15	8.05
Deep	0.29	1.78	5.36

Thus, a comparative analysis of the three volcanoes revealed differences in the composition of microbial communities. Kmv1 was characterized by a sharp transition from a surface assemblage of *Bathyarchaeia, Desulfobacterota*, and *Pseudomonadota* to a deep-level dominance of ANME-2d. At Kmv2, the surface horizons were characterized by a high share of *Chloroflexota*, while deeper horizons form an AOM zone (ANME-2a/2b, ANME-2d, and ANME-3 groups).

The high abundance of *Bathyarchaeia* in Kmv1 and Kmv2 volcanoes, particularly in the upper and middle horizons, reflects their key role in the carbon cycle, including participation in acetogenesis, fermentation, and degradation of organic compounds. Recent metagenomic studies have confirmed that members of this lineage contain the complete Wood-Ljungdahl pathway, as well as enzymes that facilitate the utilization of aromatic and high-molecular-weight compounds (Ruiz-Blas et al., 2024). *Bathyarchaeia* have been shown to be adaptable to conditions of reduced redox potential and to participate, together with other microorganisms, in anaerobic carbon metabolism (Yi et al., 2024). These data are consistent with our findings of a significant proportion of *Bathyarchaeia* in the surface horizons, where acetogenic and enzymatic pathways for organic matter processing are likely realized.

The high abundance of *Chloroflexota* in the upper and middle horizons of Kmv1 and Kmv2 volcanoes is likely due to their role in the anaerobic degradation of organic matter. Representatives of this phylum possess a wide range of enzymes associated with the decomposition of polysaccharides, fatty acids, and other organic compounds, accompanied by the formation of reduced metabolites such as hydrogen, acetate, and low-molecular-weight organic acids (Liu et al., 2022; Freches and Fradinho, 2024). These compounds can serve as substrates for methanogens and sulfate-reducing bacteria. *Chloroflexota* are actively involved in carbon remineralization in deep sediments and runoff biofilms (Petriglieri et al., 2023), and their evolutionary diversity and ability to metabolically adapt to anaerobic environments were demonstrated (Wiegand et al., 2023). Thus, the high relative abundance of *Chloroflexota* at depths of 20–70 cm apparently reflects an increase in metabolic activity in the anaerobic degradation of organic matter, resulting in the formation of reduced substrates (H_2_, acetate and low molecular weight organic acids), which, in turn, can serve as donors for methanogenesis.

Overall, the structure of the studied microbial communities in Kmv1 and Kmv2 volcanoes reflects a transition from surface microbial assemblages dominated by organotrophic microorganisms to deeper consortia including methanogens and syntrophic associations of sulfate-reducing bacteria and anaerobic methanotrophs. The results confirm the close relationship between microbial processes and environmental geochemical gradients and highlight their key role in the functioning of the methane and sulfur cycles in the mud volcanoes of the Kerch region.

Kmv3 volcano differs from the other two in that there was no clear depth-dependent pattern of community composition. It was characterized by a high abundance of ANME-3 and the presence of methanotrophic *Methylomonadaceae* bacteria and methanogenic *Methanocalculus* archaea throughout its depths, indicating the heterogeneity of this system, in which methanogenesis and methane oxidation, both aerobic and anaerobic, can occur in different microniches. This may be due to the efficient mixing of fluid in the volcanic channel, caused by the gas bubbling. A similar pattern of co-occurrence of ANME and *Methylomonadaceae* was observed in a highly bioturbated submarine methane seep (Liang et al., 2026).

The obtained results are generally consistent with data on microbial communities of mud volcanoes and methane seeps from various geographic regions, where the co-occurrence of methanogenic archaea, anaerobic methanotrophs, sulfate-reducing bacteria, and anaerobic organoheterotrophs was commonly observed (Boetius et al., 2000; Knittel and Boetius, 2009; Wegener et al., 2016; Ruff, 2020). Such consortia are considered key elements of the biogeochemical cycles of carbon and sulfur in deep-sea and subsurface ecosystems.

### MAGs of dominant microbial lineages

To analyze genomes of the most abundant or functionally important members of the microbial community, metagenomes of three fluid samples from the deepest depths were sequenced using the Illumina HiSeq 2500 platform. A total of 97 Gb of metagenomic sequences (29–36 Gb per sample) were obtained and then assembled into contigs. These contigs were binned into MAGs, of which 88 genomes had more than 90% completeness and less than 5% contamination, as assessed using CheckM2 (Supplementary Table S1).

A taxonomic classification of MAGs based on the GTDB database revealed the same major prokaryotic lineages identified by 16S rRNA gene sequencing. To gain a more detailed understanding of the metabolic capabilities of microbial lineages that were most abundant in the microbiome and/or may be involved in important processes, a more detailed analysis of several MAGs was conducted.

#### Methanogens

Among the methanogenic archaea, only one MAG was assembled, KBul-A03, found in Kmv3 and comprising approximately 0.5% of its metagenome. Taxonomically, it belongs to the genus *Methanocalculus* (family *Methanocorpusculaceae*), whose members are hydrogenotrophic methanogens (Ollivier et al., 1998). A nearly complete set of key genes for CO₂ reduction to CH₄, typical for hydrogenotrophic methanogenesis in cytochrome-deficient archaea (Thauer et al., 2008), was identified in the genome, including *fwd/fmd, ftr, mch, mer, mtrBCDEH, mcrABGCD/A2*, as well as *hdr*, several *fdh* genes, and the Eha membrane complex. Canonical genes required for methanogenesis from methanol and methylamines (*mtaBC, mtmBC, mtbBC, mttBC*) were absent. Overall, KBul-A03 archaeon is a hydrogenotrophic methanogen, likely utilizing hydrogen produced by fermentative microorganisms. Despite its low relative abundance, this organism likely contributes to the formation of the methane pool in the mud volcano.

#### ANME

MAGs of representatives of several archaeal ANME lineages were obtained from the metagenome, including ANME-1 (KBul-A09), ANME-2d (*Ca.* Methanoperedenaceae, KBul-A06 and KBul-A07), ANME-2a/2b (*Ca.* Methanocomedenaceae, KBul-A04 and KBul-A05), and ANME-3 (*Ca.* Methanovorans, KBul-A08). Analysis of these MAGs indicated the presence of a key metabolic pathway for anaerobic methane oxidation, the so-called reverse methanogenesis (Supplementary Table S2). In this process, methane is activated by the methyl-coenzyme M reductase enzyme (the Mcr complex), after which the carbon passes through a sequence of reactions of the central C1 metabolism of methanogenesis in the reverse direction (Knittel and Boetius, 2009; Evans et al., 2019; Borrel et al., 2016). Despite the universality of the central metabolic pathway of anaerobic methane oxidation, different ANME clades have their own genetic characteristics.

MAG KBul-A09, representing the ANME-1 clade (family *Methanospirareceae*, class *Syntropharchaeia* in the GTDB system), contained a complete set of genes encoding reverse methanogenesis enzymes, including the subunits of methyl-coenzyme M reductase (*mcrAGBC*), components of the Hdr system, and the Fwd/Fmd and Mtr complexes. This architecture is characteristic of ANME-1 representatives and reflects their evolutionary proximity to methanogens with a functional reorientation of metabolism toward methane oxidation (Knittel and Boetius, 2009; Borrel et al., 2016).

The ANME-2d clade (family *Ca.* Methanoperedenaceae) was represented by MAGs KBul-A06 and KBul-A07, which were assigned to the candidate genus Methanoperedens_A in the GTDB system. Both genomes contained a complete set of enzymes for reverse methanogenesis, and their distinctive feature was the presence of genes for multiheme c-type cytochromes. The presence of such proteins is considered a characteristic genomic feature of *Ca.* Methanoperedenaceae and is associated with their ability to perform extracellular electron transfer (EET). This mechanism allows archaea to transfer reducing equivalents directly to external electron acceptors, including iron and manganese oxides, or to syntrophic partners within microbial consortia (Haroon et al., 2013; Leu et al., 2020; Ouboter et al., 2024). Ecological studies have shown that ANME-2d are capable of anaerobically oxidizing methane using nitrate, nitrite, or metals as terminal electron acceptors, which distinguishes them from most marine ANME clusters, which are associated primarily with sulfate reduction (Haroon et al., 2013; Leu et al., 2020). The absence of nitrate reductase genes in the KBul-A06 and KBul-A07 genomes suggests that, unlike some other *Ca.* Methanoperedenaceae, such as *Ca.* Methanoperedens nitroreducens, the organisms we found cannot couple methane oxidation with nitrate reduction and most likely use metal oxides as electron acceptors. Thus, the dominance of ANME-2d in the Kmv1 sample (KBul-A06 represented 42% of its metagenome) may reflect conditions favorable for metal-dependent methane oxidation. Interestingly, the KBul-A06 genome contained a set of genes encoding molybdenum-iron nitrogenase, suggesting nitrogen fixation capabilities.

The ANME-2a/2b clade (family *Ca.* Methanocomedenaceae) was represented by MAGs KBul-A04 and KBul-A05. These genomes also possessed complete set of genes for reverse methanogenesis enzymes and genes encoding multiheme c-type cytochromes, indicating advanced EET capabilities. It was recently shown that members of the *Methanocomedenaceae* belonging to the ANME-2a lineage often form consortia with sulfate-reducing bacteria, implementing a classic variant of anaerobic methane oxidation coupled with sulfate reduction (Chadwick et al., 2022; Yu et al., 2022).

Of particular interest is the ANME-3 lineage, which was most abundant in the Kmv3 mud volcano, where the MAG of its representative, KBul-A08, accounted for about 7% of the entire metagenome. In the GTDB, it belongs to the candidate genus Methanovorans of the family *Methanosarcinaceae*. This MAG encoded a full set of reverse methanogenesis enzymes, including the Mcr, Hdr, and Mtr complexes, as well as a number of multiheme c-type cytochromes, which is consistent with the idea of an important role of EET-dependent processes in ANME-3 energetics. Recent studies indicate that *Ca.* Methanovorans form a separate evolutionary lineage of anaerobic methanotrophs and occupy specific ecological niches in methane-saturated sediments, where they can participate in syntrophic interactions with sulfate-reducing bacterial partners (Ouboter et al., 2024; Chadwick et al., 2022; Woods et al., 2025), although some studies suggested the possibility of independent AOM by ANME-3 (Gautam, 2018).

The observed distribution of ANME clades across the three mud volcanoes likely reflects the existence of multiple overlapping metabolic niches associated with different electron acceptors and local redox potential gradients. The dominance of ANME-2d in Kmv1 may indicate conditions favorable for metal-dependent methane oxidation, while the increased abundance of ANME-2a/2b in Kmv2 and ANME-3 in Kmv3 may reflect more classical conditions for AOM coupled with bacterial sulfate reduction. This stratification of methanotrophic communities is consistent with current understanding of microbial control of methane fluxes in anaerobic sedimentary ecosystems, where different ANME groups occupy specific ecological niches along gradients of methane and electron acceptors (Knittel and Boetius, 2009; Evans et al., 2019; Borrel et al., 2016).

#### Sulfate-reducing bacteria

A search for dissimilatory sulfate reduction pathway genes in high-quality MAGs identified 13 putative sulfate reducers (Supplementary Table S1), of which one represented the phylum *Actinomycetota* (class *Thermoleophilia*), one represented the phylum *Bacteroidota* (order *Kapaibacteriales*), one represented the phylum *Nitrospirota* (order *Thermodesulfovibrionales*), and 10 represented the phylum *Desulfobacterota* (orders *Desulfobacterales*, *Desulfobulbales*, and *Desulfatiglandales* in the GTDB system).

In the Kmv1 volcano sulfate-reducing MAGs constituted only 0.5% of the metagenome and were primarily represented by the uncultured families SURF-16 and UBA5123 of the order *Desulfobulbales*. The low abundance of sulfate reducers is consistent with the observation that the ANME community was dominated by members of *Ca.* Methanoperedenaceae, which can perform methane oxidation without syntrophic associations with sulfate-reducing bacteria.

In the Kmv3 volcano, the proportion of sulfate-reducing MAGs in the metagenome was about 2%. The most numerous groups were *Desulfobulbales* (family JAGXFP01, 1.35% of the metagenome), *Desulfobacterales* (genus *Ca. Desulfaltia*, 0.3%), and representatives of the uncultured family UBA2268 of the phylum *Bacteroidota*, order *Kapaibacteriales* (0.3%). *Desulfobulbales* are known as syntrophic partners for the ANME-3 archaea (Cassarini et al., 2019; Woods et al., 2025) dominant in Kmv3. Although *Ca.* Desulfaltia has been reported as a synthophic partner for ANME-1 archaea (Mayr et al., 2025), its ability to associate with other ANME lineages is also plausible. The third likely sulfate-reducing lineage, *Kapaibacteriales*, was found only in the Kmv3 volcano. These bacteria were previously discovered in deep groundwater and thermal springs, where their putative role was the degradation of low-molecular-weight organic compounds (Kadnikov et al., 2020). They have not been reported in mud volcano microbiomes but it is possible that members of this lineage may act as syntrophic partners for ANME-3 archaea.

Sulfate-reducing bacteria were the most abundant (7.2% of the metagenome) and diverse in the Kmv2 sample, which also harbored various ANME groups, including ANME-2a/2b, *Ca.* Methanoperedenaceae (ANME-2d), and ANME-3. The higher abundance of sulfate reducers is consistent with an order of magnitude higher sulfate concentration at this volcano (Table 2). The dominant phylotype (4.4%) was the ETH-SRB1 family of the order *Desulfobacterales*, whose members were previously described as associates of ethane-oxidizing archaea from a marine hydrocarbon seep belonging to the ANME-2d clade (Chen et al., 2019). Later, the ETH-SRB1 lineage was shown to associate with methanotrophic *Ca.* Methanoperedenaceae in the deep terrestrial subsurface (Bell et al., 2022). Overall, MAGs of the order *Desulfobacterales* accounted for 4.7% of the metagenome.

About 2% of the metagenome were representatives of the order *Desulfobulbales*, which can partner with various archaeal ANME lineages, including ANME-2a/2b (Adepoju et al., 2026). A small fraction (0.12%) of the metagenome consisted of representatives of the order *Desulfatiglandales*. *Desulfatiglandales* can form syntrophic associations with ANME archaea, especially ANME-1, and were frequently found in the sulfate–methane transition zone (SMTZ), where their presence coincides with ANME-1 abundance peaks (Kevorkian et al., 2021). About 0.2% of the metagenome consisted of sulfate reducers of the order *Thermodesulfovibrionales*, phylum *Nitrospirota*, which were frequently found in deep subsurface ecosystems (Frank et al., 2016), and were previously detected in the mud volcano of the Kerch Peninsula (Mardanov et al., 2020). Their ability to form associations with ANME archaea has not been reported.

Another group of potential sulfate reducers, detected in small amounts in Kmv1 and Kmv2 samples, were *Actinomycetota* of the class *Thermoleophilia*, representing the uncultured order and family BMS3ABIN01. The sulfate-reducing capacity of this *Actinomycetota* lineage was predicted based on genomic analysis (Diao et al., 2023) but data on their ability to act as partners of ANME archaea are lacking.

Along with sulfate-reducing bacteria, members of the genus *Desulfurivibrio* were detected in the metagenomes of mud volcanoes. Cultivated species of this genus, some of which were isolated from mud volcanoes, were capable of thriving on sulfur disproportionation (Sorokin et al., 2008; Khomyakova et al., 2026).

#### Sulfur oxidizing bacteria

A search for microorganisms capable of oxidizing sulfur and its reduced compounds identified 14 MAGs containing KEGG modules M00595 and M00985. Two MAGs represented *Campylobacterota* (family *Sulfurimonadaceae*), one—*Alphaproteobacteria* (*Roseovarius autotrophicus*), and 11—*Gammaproteobacteria* (Supplementary Table S1). Together, these MAGs constituted approximately 26% of the Kmv2 metagenome and only approximately 1% of the Kmv1 and Kmv3 metagenomes.

In the Kmv2 metagenome, MAG KBul-B66, assigned to the genus *Thiobacillus*, dominated among sulfur-oxidizing bacteria accounting for approximately 20% of the whole metagenome. Analysis of this genome revealed the presence of a sulfur metabolism system characteristic of sulfur-oxidizing chemolithotrophic bacteria. In particular, a set of Sox system genes was identified, including *soxX*, *soxA*, *soxY*, *soxZ*, and *soxB*, encoding key components of the periplasmic pathway for thiosulfate oxidation to sulfate (Ghosh and Dam, 2009; Zhou et al., 2025). Oxidation of sulfide to elemental sulfur in MAG KBul-B66 could be mediated by flavocytochrome c sulfide dehydrogenase. The detected heterodisulfide reductase complex HdrABC is probably involved in electron transfer during the oxidation of reduced sulfur compounds (Dahl, 2017; Koch and Dahl, 2018). The KBul-B66 genome also contained key genes of the reverse Dsr pathway (dissimilatory sulfite reductase) of sulfur metabolism, used by sulfur-oxidizing bacteria to oxidize intracellular elemental sulfur to sulfite (Pott and Dahl, 1998; Dahl, 2008). The adenylylsulfate reductase (*aprAB*) and sulfate adenylyltransferase (*sat*) genes, which enables the oxidation of sulfite to sulfate, were also found. A similar organization of sulfur metabolism is characteristic of representatives of *Thiobacillus* (Beller et al., 2006).

The aerobic respiratory system of MAG KBul-B66 appears to be adapted to microaerobic conditions and is characterized by the presence of the *cbb3*-type cytochrome c oxidase, which has a high affinity for oxygen and supports respiration at low oxygen concentrations (Durand et al., 2018), and cytochrome *bd* oxidase, which is characteristic of respiration at extremely low O_2_ concentrations (Borisov et al., 2021). In addition to aerobic respiration, a set of genes associated with the dissimilatory reduction of nitrogen compounds was identified in the KBul-B66 genome. In particular, genes encoding nitrate transporters and the denitrification pathway from nitrate to nitrous oxide (periplasmic nitrate reductase *napABDHG,* nitrite reductase *nirBD*, and nitric oxide reductase), were found. However, the absence of the *nosZ* gene encoding N_2_O reductase suggests that the denitrification process is incomplete and likely results in the formation of nitrous oxide.

The presence of a versatile respiratory chain and electron transport systems in the KBul-B66 bacterium allows for the efficient integration of sulfur, nitrogen, and carbon metabolism. Electrons generated during the oxidation of reduced sulfur compounds can enter the respiratory chain and be used for both aerobic and anaerobic respiration, providing the microorganism with energy flexibility in the face of changing environmental oxidation–reduction potential.

Components of the Sox system, as well as additional proteins involved in the transformation of sulfur intermediates, were identified in the genomes of other potential sulfur-oxidizing bacteria. However, some of the MAGs contained genes encoding enzymes that oxidize a wider range of sulfur compounds. For example, broad metabolic potential for sulfur compound oxidation was revealed in MAG KBul-B71, assigned to *Thioalkalivibrio thiocyanodenitrificans*. In addition to the Sox system and sulfite oxidation genes, MAG KBul-B71 also contains a thiocyanate hydrolase gene cluster, indicating the possibility of thiocyanate oxidation.

#### Archaea of the candidate phylum EX4484-52

Sequencing of mud volcano metagenomes yielded a high-quality MAG KBul-A02 (97.31% completeness, 0.5% contamination) of archaea belonging to the candidate phylum EX4484-52, also known as *Terrarchaeota* (Hamm et al., 2026) in the SeqCode Registry, a member of the DPANN group most closely related to *Nanohaloarchaeota* (Zhao et al., 2022; Hamm et al., 2026). This phylotype was present only in Kmv1 and comprised approximately 0.8% of its metagenome. Because KBul-A02 was the best quality (highest completeness) of all known EX4484-52 MAGs, we analyzed the KBul-A02 genome to elucidate its phylogenetic position, the metabolic capabilities, lifestyle, and ecological role of these archaea.

**Figure 5:**
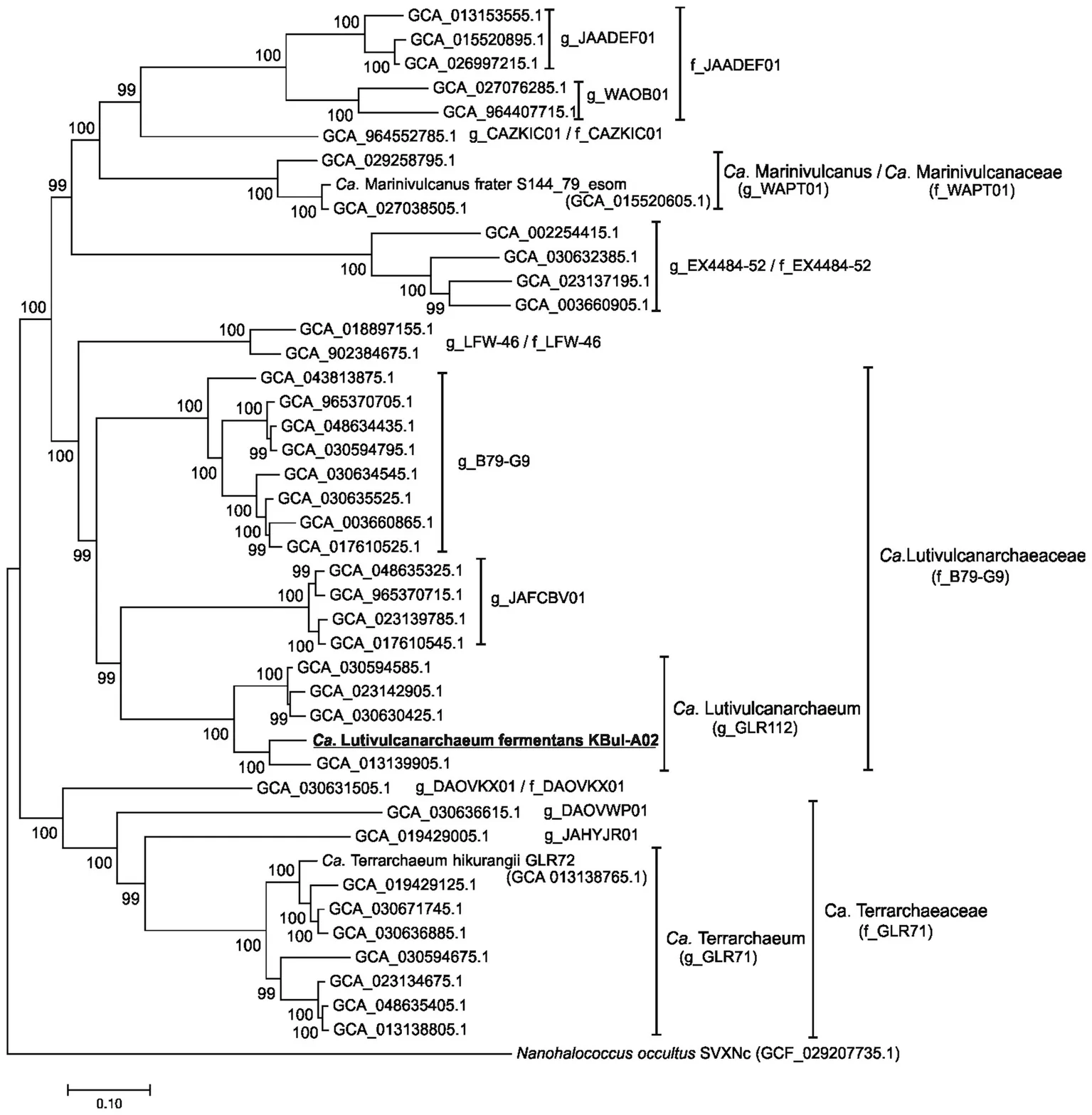
Genome-based phylogeny of the candidate phylum EX4484-52. Taxonomic lineages are shown according to the GTDB release 232 (g, genus; f, family). The level of support for internal branches was calculated using the Bayesian test. The genome sequence of *Nanohalococcus occultus*, a member of the sister phylum *Nanohalarchaeota* was used as an outgroup.

The KBul-A02 genome is 1,266,221 nt in size and, according to the GTDB classification, belongs to the class EX4484-52, order EX4484-52, family B79-G9, and genus GLR112. To further characterize the phylogenetic position of KBul-A02, a maximum likelihood phylogenetic tree based on 120 conserved genes was constructed for this MAG and representative genomes from all other species-level lineages (1 genome per species) of the EX4484-52 phylum. All genera and families defined in the GTDB within the EX4484-52 phylum formed distinct branches (Figure 5), including previously proposed *Ca.* Marinivulcanus and *Ca.* Terrarchaeum (Hamm et al., 2026). The closest relatives of KBul-A02 were four MAGs assigned to the genus GLR112 (Figure 5). KBul-A02 share 72.5–79.8% ANI with these genomes, and can therefore be classified as a new species in this genus.

**Figure 6:**
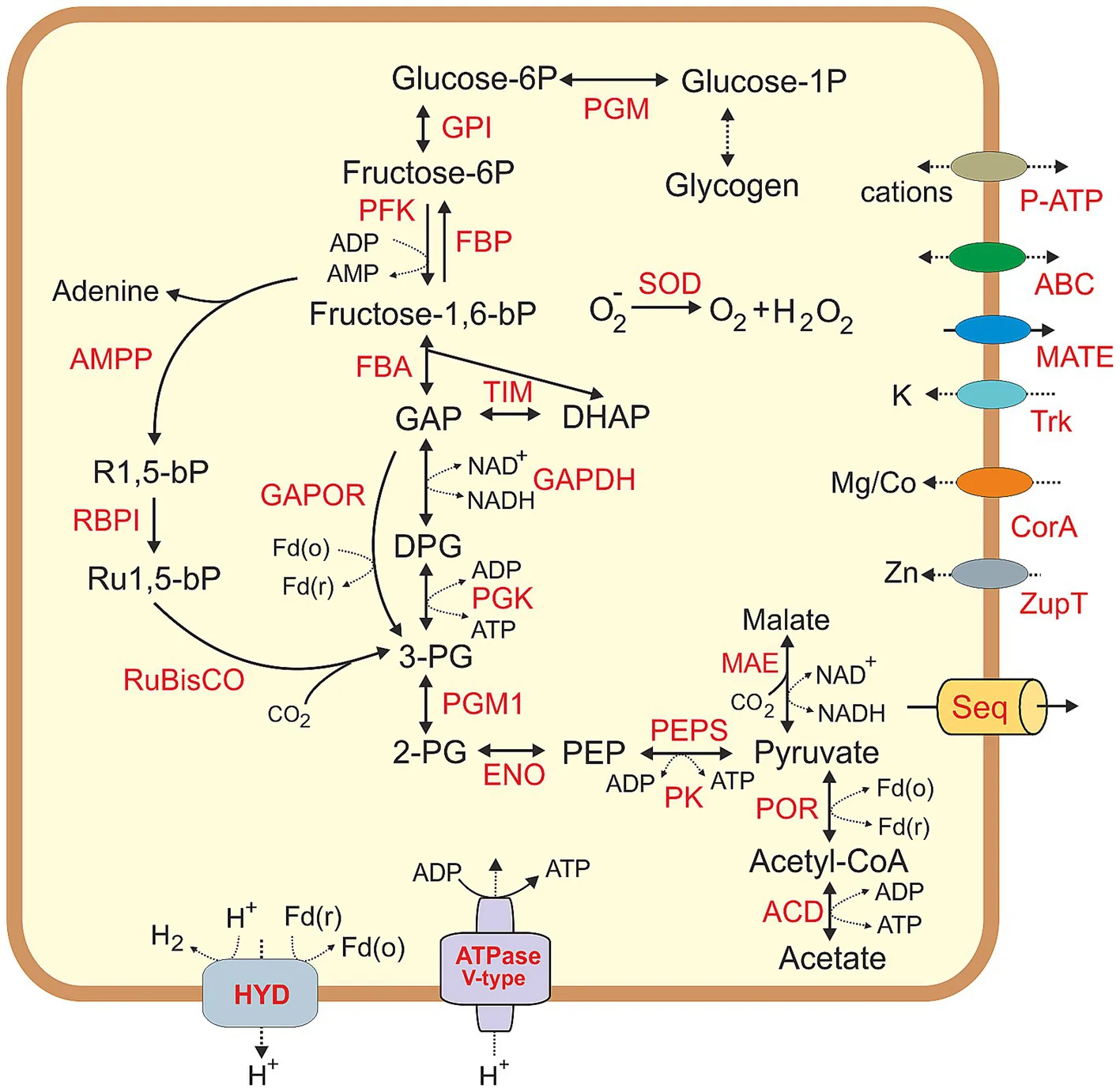
Overview of catabolic pathways encoded by the KBul-A02 genome. Enzyme abbreviations: PGM, phosphoglucomutase; GPI, glucose-6-phosphate isomerase; PFK, ADP-dependent phosphofructokinase; FBP, fructose-1,6-bisphosphatase; FBA, fructose-1,6-bisphosphate aldolase; TIM, triosephosphate isomerase; GAPOR, glyceraldehyde-3-phosphate:ferredoxin oxidoreductase; GAPDH, glyceraldehyde-3-phosphate dehydrogenase; PGK, 3-phosphoglycerate kinase; PGM1, phosphoglycerate mutase; ENO, enolase; PK, pyruvate kinase; PEPS, phosphoenolpyruvate synthase; POR, pyruvate:ferredoxin oxidoreductase; ACD, acetyl-CoA synthetase; MAE, malic enzyme; AMPP, AMP phosphorylase; RBPI, ribose-1,5-bisphosphate isomerase; RuBisCO, ribulose-1,5-bisphosphate carboxylase; SOD, superoxide dismutase; HYD, membrane-linked hydrogenase complex; P-ATP, P-type ATPase; ABC, ABC-type transporter. Compounds abbreviations: GAP, glyceraldehyde-3-phosphate; DHAP, dihydroxyacetone phosphate; DPG, 2,3-diphospho-glycerate; 3-PG, 3-phosphoglycerate; 2-PG, 2-phosphoglycerate; PEP, phosphoenolpyruvate; R1,5-bP, ribose 1,5-bisphosphate; Ru1,5-bP, ribulose 1,5-bisphosphate; Fd(ox), oxidized ferredoxin; Fd(red), reduced ferredoxin.

The central carbon metabolism of KBul-A02 (Figure 6) is represented by a modified version of the Embden–Meyerhof pathway, typical of hyperthermophilic anaerobic archaea. Characteristic markers of this pathway are ADP-dependent phosphofructokinase and glyceraldehyde-3-phosphate:ferredoxin oxidoreductase (GAPOR), present alongside the classical phosphorylating glyceraldehyde-3-phosphate dehydrogenase (GAPDH), indicating parallel functioning of the ferredoxin-dependent and NAD-dependent branches of glycolysis (Tuininga et al., 1999; van der Oost et al., 1998; Bräsen et al., 2014). Enzymes specific for gluconeogenesis, namely, phosphoenolpyruvate synthase and fructose-1,6-bisphosphatase, are encoded as well.

Pyruvate oxidation to acetyl-CoA can be carried out by ferredoxin-dependent pyruvate:ferredoxin oxidoreductase, and acetyl-CoA metabolism is provided by acetyl-CoA synthetase and ADP-forming acetate-CoA ligase. The tricarboxylic acid cycle and aerobic or anaerobic respiration pathways are absent, as well as autotrophic carbon fixation pathways, indicating an anaerobic fermentative metabolism (Adams et al., 2001; Bräsen et al., 2014). Glycogen can act as a storage polysaccharide, as evidenced by the presence of genes involved in its metabolism, including GH57 family alpha-amylase, glycogen synthase, and glycogen debranching enzyme.

Interestingly, the KBul-A02 genome contains a gene encoding ribulose-1,5-bisphosphate carboxylase type IIIb (RuBisCO). These enzymes in Archaea are known to be involved in the AMP salvage pathway rather than in the Calvin cycle (Sato et al., 2007). Other genes of this pathway, AMP phosphorylase and ribose-1,5-bisphosphate isomerase, are also present suggesting the ability to utilize AMP. Sufficient amounts of AMP could be formed, for example, in glycolysis by ADP-dependent phosphofructokinase.

A key element of KBul-A02 energy metabolism is the MBH/Mrp-like hydrogenase module coupled to transmembrane ion transport. Maturation factors for [NiFe]-hydrogenases, as well as components of a multisubunit ion-transporting hydrogenase, have been identified in the genome. Its organization corresponds to the modular MBH/Mrp complexes described for *Thermococcales*, in which the oxidation of reduced ferredoxin is coupled to hydrogen formation and the generation of a proton/sodium gradient (Sapra et al., 2003; Schut et al., 2013; Yu et al., 2018). The energy of this gradient could be used for ATP synthesis via A/V-type ATP synthase. MBS/NSR sulfur reductase systems used by hyperthermophilic archaea to reduce elemental sulfur were not identified in the genome (Muhlbauer et al., 2021; Pisa et al., 2007; Kanai et al., 2011).

The search for hydrolytic enzymes did not reveal proteases and glycosyl hydrolases containing N-terminal secretion signals that could enable the extracellular hydrolysis of complex biopolymers. Canonical transporters for the importation of sugars and amino acids/peptides were not identified, suggesting narrow substrate specialization of KBul-A02 bacterium, or its reliance on acquisition of metabolites from partner cells within symbiotic or parasitic lifestyle, as proposed for other members of DPANN (Dombrowski et al., 2019; Wurch et al., 2016). Analysis of the KBul-A02 genome revealed the absence or incompleteness of many biosynthetic pathways for key cellular compounds, including amino acids, vitamins, nucleotides, thiamine, various cofactors, and lipids, as observed in many other DPANN lineages (Dombrowski et al., 2019).

Thus, KBul-A02 represented a strictly anaerobic archaea with a fermentative metabolism. Energy metabolism is carried out through ferredoxin-dependent reactions, resulting in the accumulation of reduced ferredoxin; its reoxidation is coupled to the formation of molecular hydrogen and the transport of H^+^/Na^+^ ions across the membrane, and the resulting transmembrane ion gradient is used by A/V-type ATP synthase for ATP synthesis (Adams et al., 2001; Yokooji et al., 2013; Williams et al., 2025). The absence of metabolic pathways for the synthesis of many key cellular components indicates a partner-dependent parasitic or symbiotic lifestyle.

The GTDB (release 232) contains 55 genomes assigned to the candidate phylum EX4484-52 (Supplementary Table S3). Most of these MAGs were retrieved from marine sediments (45 MAGs), including such sites as cold seeps and hydrothermal vents; other MAGs were obtained from metagenomes of deep groundwater (8 MAGs) and mud volcano fluids in the Taman region (2 MAGs). Thus, this lineage appears to be indigenous to the deep subsurface biosphere.

In order to obtain information about the lifestyle of EX4484-52 bacteria, we conducted a comparative genomic analysis of its representatives and analyzed the presence of important metabolic pathways using the DRAM tool (Supplementary Figure S1). Since the absence or incompleteness of particular genes/pathways may result from the incompleteness of the assembly, only 13 MAGs with more than 90% completeness from GTDB and KBul-A02 were included in this analysis. All these MAGs appeared to possess a nearly complete Embden-Meyerhof pathway, but lacked the tricarboxylic acid cycle and respiratory functions, as well as the autotrophic carbon fixation pathways, indicating anaerobic fermentative metabolism. Interestingly, most of the MAGs possess type A/V ATP synthase, which could allow the use of a transmembrane ion gradient for ATP synthesis.

Taking into account that the KBul-A02 MAG is the best quality genome of a member of the candidate phylum EX4484-52, and that it meets the criteria for a high-quality MAG (Bowers et al., 2017) and considering the recently published consensus statement regarding naming uncultivated Archaea and Bacteria (Murray et al., 2020) we propose the following taxonomic names for the novel family, genus, and species of KBul-A02 under the rules of the International Code of Nomenclature of Prokaryotes.

### Description of *Candidatus Lutivulcanarchaeum* gen. nov

Lu.ti.vul.can.ar.chae’um. L. neut. n. *lutum*, mud; L. masc. n. *Vulcanus*, the Roman god of fire, used here to denote an origin from mud volcano; N. L. neut. n. *archaeum*, an archaeon; N. L. neut. n. Lutivulcanarchaeum, an archaeon from a mud volcano.

### Description of *Candidatus Lutivulcanarchaeum fermentans* sp. nov

*Lutivulcanarchaeum fermentans* (fer.men’tans. L. neut. part. adj. *fermentans*, fermenting).

Not cultivated. Inferred to be an anaerobic, obligate organotroph that obtains energy through fermentation of low-molecular-weight organic substrates. Represented by the genome (acc. no to be provided) assembled from the metagenome of a terrestrial mud volcano fluid.

Based on this, we propose the following name for the family:

“*Candidatus* Lutivulcanarchaeaceae” fam. nov.

The candidate genus Lutivulcanarchaeum corresponds to the genus GLR112, while the candidate family Lutivulcanarchaeaceae corresponds to the family B79-G9 in the phylum EX4484-52 in the GTDB system.

### Comparative analysis of the genetic potential of microbial communities of mud volcanoes

**Figure 7:**
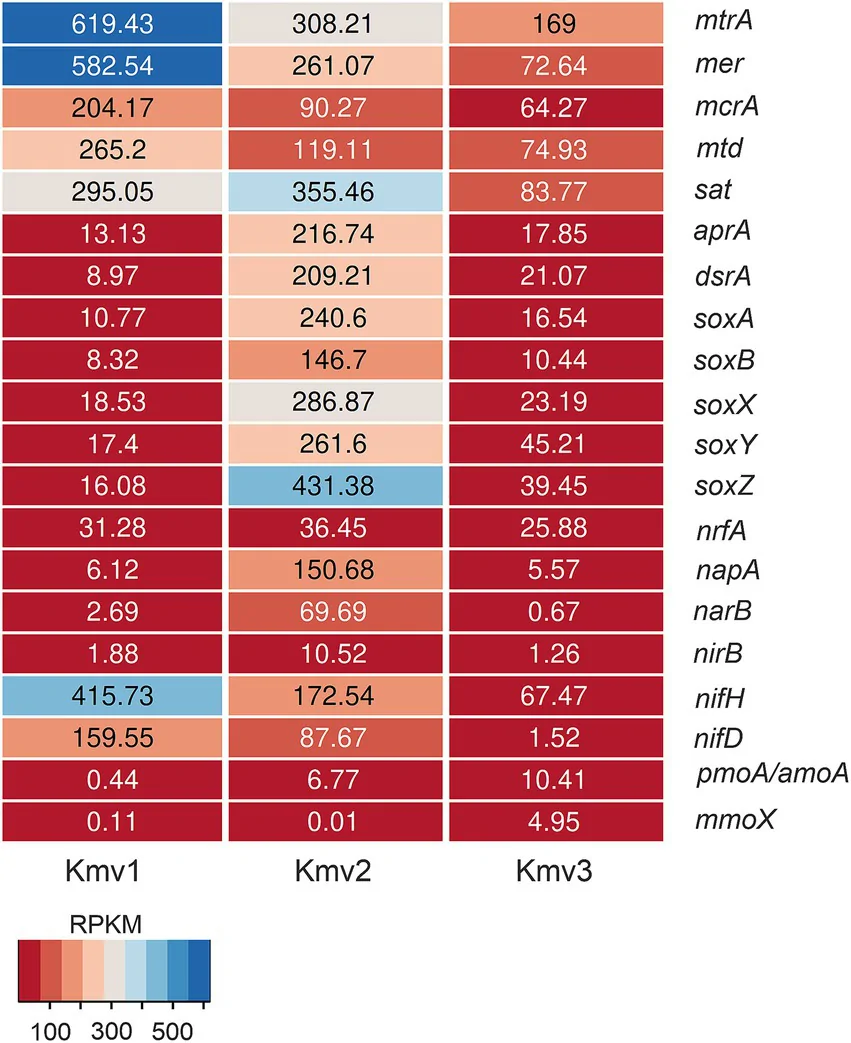
Relative abundance of key genes associated with carbon, sulfur, and nitrogen metabolism in mud volcano metagenomes (normalized gene coverage values).

To predict the importance of key pathways of methane, sulfur, and nitrogen metabolism in the three sequenced metagenomes, the relative abundance of relevant genes identified in the contigs was determined (Figure 7). Since the assembled contigs represented 92, 91, and 84% of the Kmv1_150, Kmv2_150, and Kmv3_180 metagenomes, respectively, the results of this comparison fairly fully reflect the abundance of the corresponding genes in the metagenomes.

Kmv1 volcano was characterized by the highest abundance of genes involved in the methane cycle, namely *mtrA, mer, mcrA*, and *mtd*. This volcano also exhibited a higher abundance of the *nifH* and *nifD* genes, indicating a higher potential for nitrogen fixation compared to the other two volcanoes. These patterns are consistent with the high share of ANME archaea of the family *Ca.* Methanoperedenaceae in this metagenome (44%).

Kmv2 also exhibited a high abundance of methane cycle genes, consistent with the high proportion of MAGs from various ANME archaeal lineages in its metagenome (approximately 20% of the metagenome). Kmv2 volcano was characterized by the highest abundance of sulfur cycle genes, including both dissimilatory sulfate reduction (*sat, aprA, dsrA*) and oxidation of reduced sulfur compounds (*soxABXYZ*). These data are consistent with the high abundance of MAGs sulfate reducers (~10%) and sulfur-oxidizing bacteria (~26%) in this metagenome. Such combination indicates high functional plasticity of the microbial community and its ability to close the sulfur cycle within a single ecosystem (Meyer and Kuever, 2007; Zhou et al., 2025). In this metagenome, a relatively high abundance of key genes involved in dissimilatory nitrogen metabolism (*napA, narB*, and *nirB*) was observed, indicating a close relationship between the sulfur cycle and the denitrification pathways. This pattern reflects the coupling of the sulfur and nitrogen cycles, characteristic of anaerobic and mixotrophic ecosystems (Kraft et al., 2011; Anantharaman et al., 2018).

Kmv3 volcano showed the lowest abundance of methanogenesis genes, consistent with the low proportion of MAGs of ANME archaea in its metagenome (approximately 8%). Sulfur cycle gene abundance was moderate, while nitrogen metabolism genes were poorly represented, consistent with microbial community composition data. Sample Kmv3 had the highest abundance of methane monoxygenase genes (*pmoA/amoA* and *mmoX*), indicating the important role of aerobic methane oxidation and consistent with the presence of methanotrophs of the family *Methylomonadaceae*.

Overall, significant differences in the content of genes involved in the methane, sulfur, and nitrogen biogeochemical cycles were identified among the studied mud volcano metagenomes. Taken together, the obtained data support the concept of a mosaic microbial community structure, in which different niches reflect the dominance of distinct, yet functionally interconnected, biogeochemical processes.

### Common genotypes of microorganisms in mud volcanoes of the Kerch-Taman mud volcanic province

In addition to the three mud volcanoes studied in this work, metagenomics methods were previously used to investigate the microbial communities of two other mud volcanoes in the Kerch-Taman region. One of the volcanoes, located on the same Bulganak mud volcano field near the city of Kerch (45.4261 N 36.4780 E), was sampled in 2019 and described in our previous work (Mardanov et al., 2020). Unfortunately, it was inactive in 2022, preventing its reanalysis. The metagenome of another mud volcano, located further east, on the Taman Peninsula and sampled in June 2022 was reported by Slobodkin et al. (2024).

**Figure 8:**
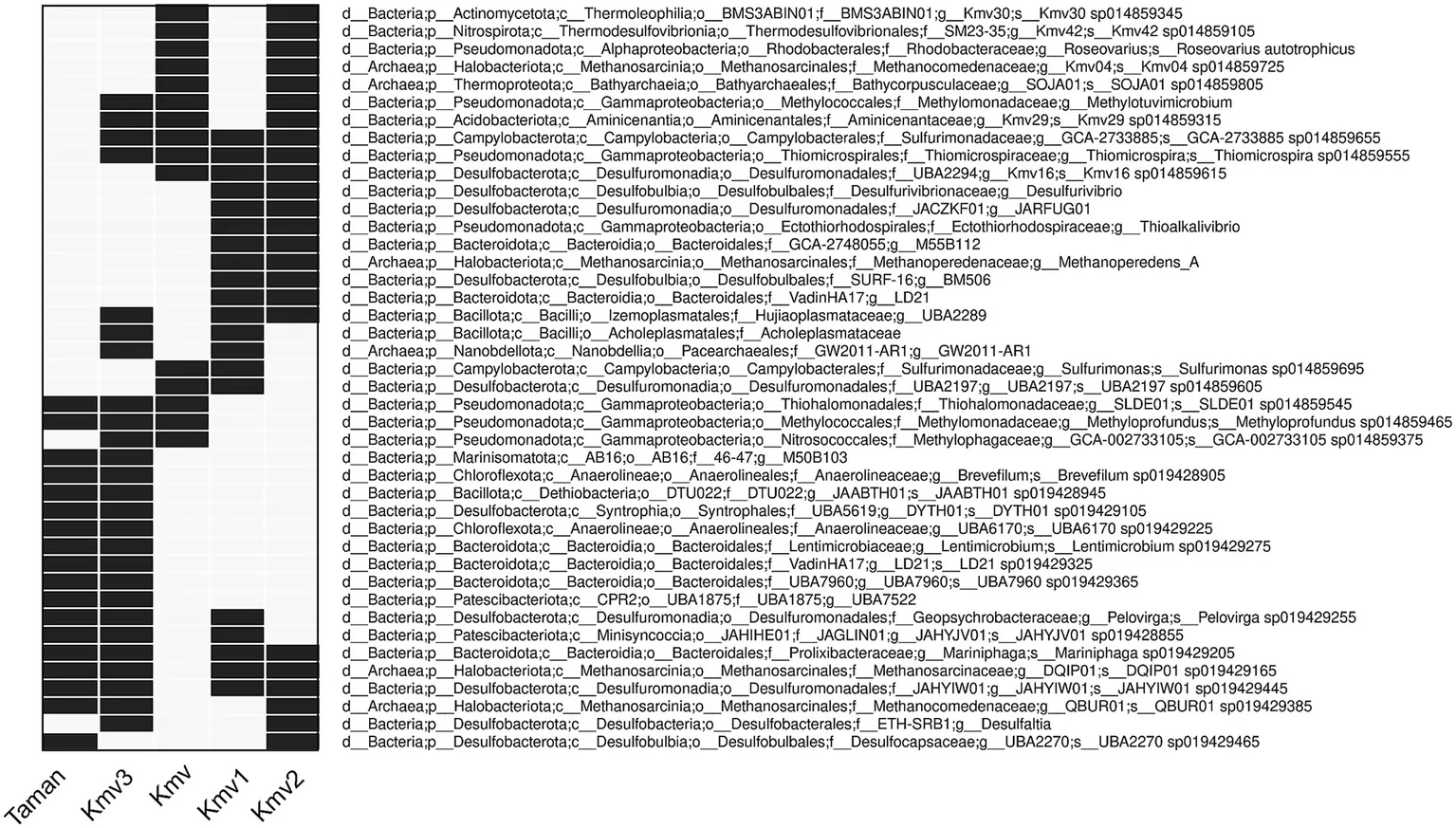
Presence of similar genotypes in mud volcanoes of the Kerch-Taman mud volcanic province. Kmv, MAGs from the metagenome of the Kerch volcano (PRJNA636628, Mardanov et al., 2020). Taman, MAGs from the metagenome of the Taman volcano (PRJNA833668, Slobodkin et al., 2024). MAGs were considered present in two or more samples if their pairwise ANI were above 97%.

A comparison of MAGs obtained from the mud volcano metagenomes revealed the presence of identical genotypes in up to four of the five sites (Figure 8). A large number of common genotypes were expectedly found in the Kmv1 and Kmv2 samples, consistent with the above data on the composition of their microbial communities. The Kmv volcano, located near Kmv2, also contained several genotypes shared with Kmv1 and especially with Kmv2.

Interestingly, many common genotypes were found in Kmv3 and a mud volcano located on the Taman Peninsula, approximately 108 km apart. In the Taman sample, the most numerous groups of anaerobic methanotrophs were ANME-2a/2b (*Ca.* Methanocomedenaceae) and ANME-3, and we also found the same lineages in Kmv3. The large number of MAGs with similar nucleotide sequences in Kmv3 and Taman suggests that microbial communities in geographically distant mud volcanoes with similar environmental and geochemical conditions may be very similar. This was also shown in the work of Tu et al. (2022) on biogeographic patterns of mud volcano microbial communities, which highlighted that community composition is determined not only by the distance between sites but also by the geochemical regime features, the availability of electron donors and acceptors, and the structure of redox gradients.

### Comparative analysis of microbial communities of terrestrial mud volcanoes from different regions of the world

To compare the compositions of microbial communities from different regions of the world, a principal coordinate analysis (PCoA) based on phylogenetically weighted UniFrac distances was performed. In addition to the nine samples described in this study, the objects of comparison included one sample from the same Bulganak mud volcano field described previously (Mardanov et al., 2020), 12 samples from mud volcanoes of the Taman Peninsula (Slobodkin et al., 2024), nine samples from Sicily, Italy (Chen et al., 2024), eight samples from Japan, and one from the Junngar Basin, China.

**Figure 9:**
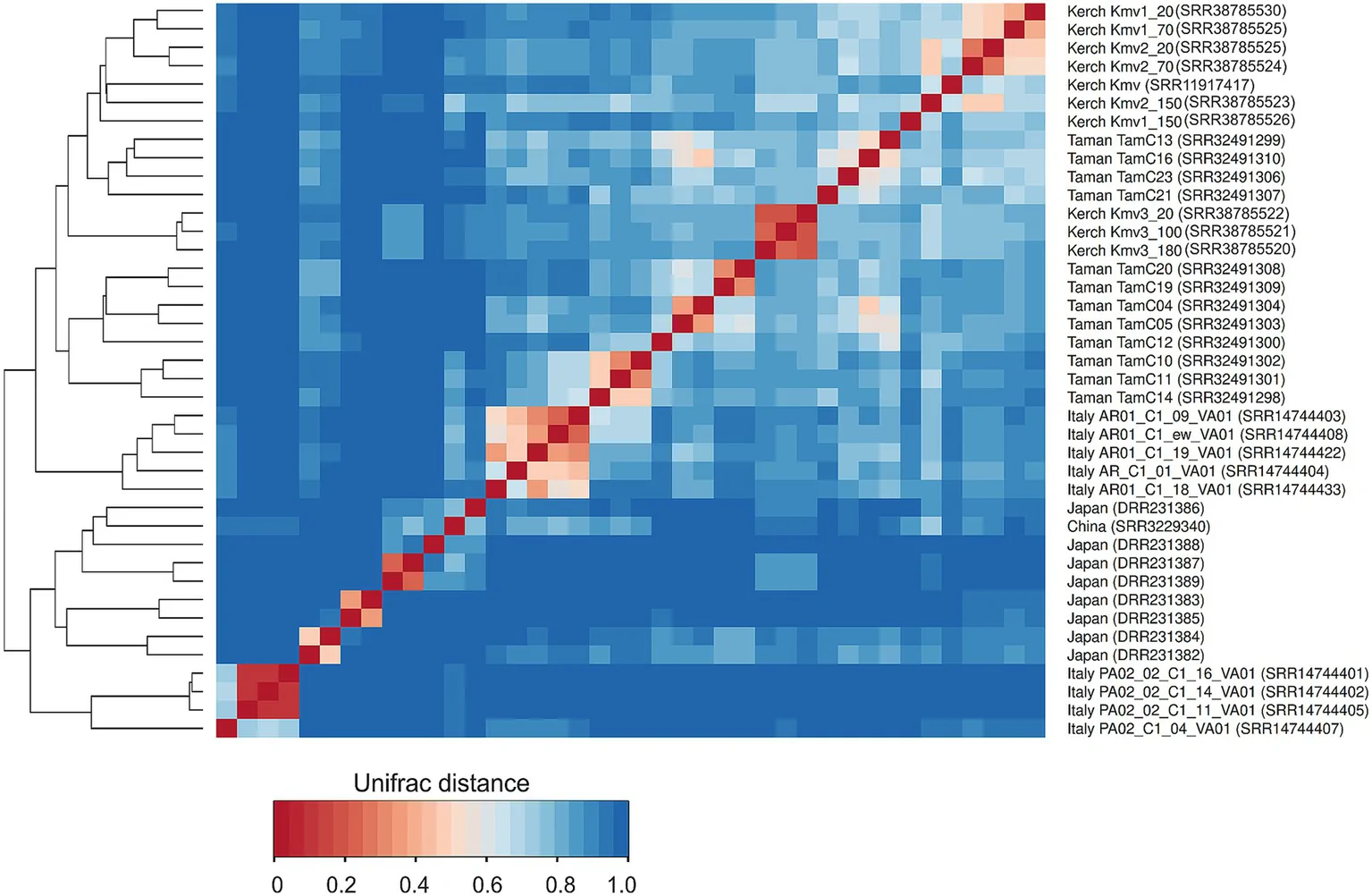
Heatmap of weighted UniFrac distances between terrestrial mud volcanoes samples with hierarchical clustering using the complete linkage method.

The obtained results show that microbial communities of mud volcanoes cluster primarily by geographic origin (Figure 9). Specific clusters were formed by samples from Japan and China, as well as the Kerch-Taman mud volcano province. In the latter case, no clear separation was observed between the Kerch and Taman samples. This pattern apparently reflects the differences in local geochemical features and the depth of fluid sampling.

Samples from Italy (Sicily) formed two distinct dense clusters. AR01 samples collected from the Maccalube di Aragona region had a chloride concentration of 200–300 mM and a pH of 7.8–8.6, while the second cluster of Sicilian volcanoes, PA02 (LeSalinelle del Vallone Salato, located near Mount Etna), had a chloride concentration of 1.2–2.1 mM and a pH of 6.3. *Woesearchaeota*, ANME-2a/2b, *Desulfobacterota, Chloroflexota*, and *Bacteroidota* predominated in AR01 samples, while *Halobacteriales*, *Gammaproteobacteria, Deferribacterota, Desulfobacterota, Chloroflexota* were most numerous lineages in PA02 samples. This division of Sicilian mud volcanoes is likely due to differences in the geochemical conditions of the habitats, primarily salinity, but also methane content and the degree of oxygenation of surface fluids, which have a significant impact on the structure of microbial communities (Chen et al., 2024).

## Conclusion

Despite the close proximity of the three studied mud volcanoes, the chemical composition of the mud fluids and their microbial communities strongly differed. The observed depth distribution of taxa, clearly visible in volcanoes Kmv1 and Kmv2, is consistent with the proposed redox stratification of the fluids. Surface horizons likely correspond to zones with higher oxidation potential and intense organic matter degradation by heterotrophic microbial communities. With increasing depth, an increase in the relative abundance of ANME archaea was observed, indicating an increased role of strictly anaerobic metabolic processes. In volcano Kmv1, deep horizons (1.5 m) were dominated by ANME archaea of the *Methanoperedenaceae* lineage, while the abundance of sulfate-reducing and sulfur-oxidizing bacteria was low. Apparently, the dominant process in this volcano was anaerobic oxidation of methane, coupled with the reduction of iron oxides, but not with nitrate or sulfate reduction.

A complex community was discovered in the deep horizon of the Kmv2 volcano, dominated by ANME archaea of the *Methanoperedenaceae* and ANME-2a/2b groups and sulfur cycle bacteria, including both sulfate-reducing and sulfur-oxidizing lineages. The metagenome of this sample also contained the highest abundance of genes associated with denitrification. Apparently, the higher sulfate content in the mud fluid of this volcano provides favorable conditions for the development of sulfur cycle microorganisms, as well as the formation of consortia between ANME-2a/2b archaea and sulfate reducers, which carry out the ANME process alongside partner-independent *Methanoperedenaceae*.

A distinctive feature of the Kmv3 volcano was the lack of a pronounced correlation between the composition of microbial communities and depth, Microorganisms involved in the methane and sulfur cycles, namely, methanogenic archaea, ANME archaea of the ANME-3 lineage, methanotrophic bacteria, sulfate reducers, and small amounts of sulfur oxidizers were simultaneously detected. Such community composition indicates the existence of localized microniches with different redox conditions and/or more efficient fluid mixing within the volcanic conduit associated with gas escaping to the surface.

## Data Availability

The sequences of 16S rRNA gene fragments, metagenome sequences and annotated sequences of MAGs have been deposited in the NCBI GenBank database and are available within the framework of the BioProject PRJNA1468499.

## References

[ref1] Adams M. W. Holden J. F. Menon A. L. Schut G. J. Grunden A. M. Hou C. . (2001). Key role for sulfur in peptide metabolism and in regulation of three hydrogenases in the hyperthermophilic archaeon *Pyrococcus furiosus*. J. Bacteriol. 183, 716–724. doi: 10.1128/jb.183.2.716-724.2001, 11133967 PMC94929

[ref2] Adepoju L. A. McKaig J. M. Salcedo R. S. Martinez A. Buessecker S. Skoog E. J. . (2026). Anaerobic methane oxidation by ANME-2a at two molar chloride in Orca Basin. bioRxiv, 2026.02.09.704686. doi: 10.64898/2026.02.09.704686

[ref3] Alizadeh A. A. Ogly Guliyev I. S. Kadirov F. A. Eppelbaum L. V. (2016). Geosciences of Azerbaijan, vol. 1 Heidelberg: Springer, 237.

[ref4] Alneberg J. Bjarnason B. S. De Bruijn I. Schirmer M. Quick J. Ijaz U. Z. . (2014). Binning metagenomic contigs by coverage and composition. Nat. Methods 11, 1144–1146. doi: 10.1038/nmeth.3103, 25218180

[ref5] Anantharaman K. Hausmann B. Jungbluth S. P. Kantor R. S. Lavy A. Warren L. A. . (2018). Expanded diversity of microbial groups that shape the dissimilatory sulfur cycle. ISME J. 12, 1715–1728. doi: 10.1038/s41396-018-0078-0, 29467397 PMC6018805

[ref6] Aramaki T. Blanc-Mathieu R. Endo H. Ohkubo K. Kanehisa M. Goto S. . (2020). KofamKOALA: KEGG Ortholog assignment based on profile HMM and adaptive score threshold. Bioinformatics 36, 2251–2252. doi: 10.1093/bioinformatics/btz859, 31742321 PMC7141845

[ref7] Aziz R. K. Bartels D. Best A. A. DeJongh M. Disz T. Edwards R. A. . (2008). The RAST server: rapid annotations using subsystems technology. BMC Genomics 9:75. doi: 10.1186/1471-2164-9-75, 18261238 PMC2265698

[ref8] Bell E. Lamminmäki T. Alneberg J. Qian C. Xiong W. Hettich R. L. . (2022). Active anaerobic methane oxidation and sulfur disproportionation in the deep terrestrial subsurface. ISME J. 16, 1583–1593. doi: 10.1038/s41396-022-01207-w, 35173296 PMC9123182

[ref9] Beller H. R. Chain P. S. Letain T. E. Chakicherla A. Larimer F. W. Richardson P. M. . (2006). The genome sequence of the obligately chemolithoautotrophic, facultatively anaerobic bacterium *Thiobacillus denitrificans*. J. Bacteriol. 188, 1473–1488. doi: 10.1128/jb.188.4.1473-1488.2006, 16452431 PMC1367237

[ref10] Boetius A. Ravenschlag K. Schubert C. J. Rickert D. Widdel F. Gieseke A. . (2000). A marine microbial consortium apparently mediating anaerobic oxidation of methane. Nature 407, 623–626. doi: 10.1038/35036572, 11034209

[ref11] Borisov V. B. Siletsky S. A. Paiardini A. Hoogewijs D. Forte E. Giuffre A. . (2021). Bacterial oxidases of the cytochrome bd family: redox enzymes of unique structure, function, and utility as drug targets. Antioxid. Redox Signal. 34, 1280–1318. doi: 10.1089/ars.2020.8039, 32924537 PMC8112716

[ref12] Borrel G. Adam P. S. Gribaldo S. (2016). Methanogenesis and the Wood-Ljungdahl pathway: An ancient, versatile, and fragile association. Genome Biol. Evol. 8, 1706–1711. doi: 10.1093/gbe/evw114, 27189979 PMC4943185

[ref13] Bowers R. M. Kyrpides N. C. Stepanauskas R. Harmon-Smith M. Doud D. Reddy T. B. K. . (2017). Minimum information about a single amplified genome (MISAG) and a metagenome-assembled genome (MIMAG) of bacteria and archaea. Nat. Biotechnol. 35, 725–731. doi: 10.1038/nbt.3893, 28787424 PMC6436528

[ref14] Brankovits D. Pohlman J. W. (2020). Methane oxidation dynamics in a karst subterranean estuary. Geochim. Cosmochim. Acta 277, 320–333. doi: 10.1016/j.gca.2020.03.007

[ref15] Bräsen C. Esser D. Rauch B. Siebers B. (2014). Carbohydrate metabolism in Archaea: current insights into unusual enzymes and pathways and their regulation. Microbiol. Mol. Biol. Rev. 78, 89–175. doi: 10.1128/mmbr.00041-13, 24600042 PMC3957730

[ref16] Brustur T. Stănescu I. Macaleţ R. Melinte-Dobrinescu M. C. (2015). The mud volcanoes from Berca: a significant geological patrimony site of the Buzău Land Geopark (Romania). GeoEcoMarina 21:73. doi: 10.5281/zenodo.45066

[ref17] Cassarini C. Zhang Y. Lens P. N. (2019). Pressure selects dominant anaerobic methanotrophic phylotype and sulfate reducing bacteria in coastal marine Lake Grevelingen sediment. Front. Environ. Sci. 6:162. doi: 10.3389/fenvs.2018.00162

[ref18] Cesar J. Mayer B. Humez P. (2021). A novel isotopic approach to distinguish primary microbial and thermogenic gases in shallow subsurface environments. Appl. Geochem. 131:105048. doi: 10.1016/j.apgeochem.2021.105048

[ref19] Chadwick G. L. Skennerton C. T. Laso-Pérez R. Leu A. O. Speth D. R. Yu H. . (2022). Comparative genomics reveals electron transfer and syntrophic mechanisms differentiating methanotrophic and methanogenic archaea. PLoS Biol. 20:e3001508. doi: 10.1371/journal.pbio.3001508, 34986141 PMC9012536

[ref20] Chaumeil P. A. Mussig A. J. Hugenholtz P. Parks D. H. (2022). GTDB-Tk v2: memory friendly classification with the genome taxonomy database. Bioinformatics 38, 5315–5316. doi: 10.1093/bioinformatics/btac672, 36218463 PMC9710552

[ref21] Chen J. N. Chiu Y. P. Tu T. H. Italiano F. Wang P. L. Lin L. H. (2024). Variations in microbial community compositions and processes imposed under contrast geochemical contexts in Sicilian mud volcanoes, Italy. Front. Microbiol. 15:1461252. doi: 10.3389/fmicb.2024.1461252, 39372275 PMC11449744

[ref22] Chen S. C. Musat N. Lechtenfeld O. J. Paschke H. Schmidt M. Said N. . (2019). Anaerobic oxidation of ethane by archaea from a marine hydrocarbon seep. Nature 568, 108–111. doi: 10.1038/s41586-019-1063-0, 30918404

[ref24] Chen B. Yu Y. Su Q. Yang L. Fu T. Liu W. . (2023). The study on the genesis of underground brine in Laizhou Bay based on hydrochemical data. Water 15:3788. doi: 10.3390/w15213788

[ref25] Cheng T. W. Chang Y. H. Tang S. L. Tseng C. H. Chiang P. W. Chang K. T. . (2012). Metabolic stratification driven by surface and subsurface interactions in a terrestrial mud volcano. ISME J. 6, 2280–2290. doi: 10.1038/ismej.2012.61, 22739492 PMC3504961

[ref26] Chklovski A. Parks D. H. Woodcroft B. J. Tyson G. W. (2023). CheckM2: a rapid, scalable and accurate tool for assessing microbial genome quality using machine learning. Nat. Methods 20, 1203–1212. doi: 10.1038/s41592-023-01940-w, 37500759

[ref27] Collado C. Romero-Tena P. Wegener G. Elvert M. Menapace W. Laso-Pérez R. (2026). Anaerobic oxidation of methane supports a minimal microbial community in a subsurface biofilm at Ginsburg mud volcano. ISME Commun. 6:ycag072. doi: 10.1093/ismeco/ycag072, 42040505 PMC13109099

[ref28] Dahl C. (2008). “Inorganic sulfur compounds as electron donors in purple sulfur bacteria,” in Sulfur Metabolism in Phototrophic Organisms, (Dordrecht: Springer Netherlands), 289–317.

[ref29] Dahl C. (2017). “Sulfur metabolism in phototrophic bacteria,” in Modern Topics in the Phototrophic Prokaryotes: Metabolism, Bioenergetics, and Omics, (Cham: Springer International Publishing), 27–66.

[ref30] Deng Y. Liang C. Zhu X. Zhu X. Chen L. Pan H. . (2024). Methylomonadaceae was the active and dominant methanotroph in Tibet lake sediments. ISME Commun. 4:ycae032. doi: 10.1093/ismeco/ycae032, 38524764 PMC10960969

[ref31] Diao M. Dyksma S. Koeksoy E. Ngugi D. K. Anantharaman K. Loy A. . (2023). Global diversity and inferred ecophysiology of microorganisms with the potential for dissimilatory sulfate/sulfite reduction. FEMS Microbiol. Rev. 47:fuad058. doi: 10.1093/femsre/fuad058, 37796897 PMC10591310

[ref32] Dimitrov L. I. (2002). Mud volcanoes—the most important pathway for degassing deeply buried sediments. Earth-Sci. Rev. 59, 49–76. doi: 10.1016/S0012-8252(02)00069-7

[ref33] Dombrowski N. Lee J. H. Williams T. A. Offre P. Spang A. (2019). Genomic diversity, lifestyles and evolutionary origins of DPANN archaea. FEMS Microbiol. Lett. 366:fnz008. doi: 10.1093/femsle/fnz008, 30629179 PMC6349945

[ref34] Durand A. Bourbon M. L. Steunou A. S. Khalfaoui-Hassani B. Legrand C. Guitton A. . (2018). Biogenesis of the bacterial cbb3 cytochrome c oxidase: active subcomplexes support a sequential assembly model. J. Biol. Chem. 293, 808–818. doi: 10.1074/jbc.M117.805184, 29150446 PMC5777255

[ref35] Edgar R. C. (2010). Search and clustering orders of magnitude faster than BLAST. Bioinformatics 26, 2460–2461. doi: 10.1093/bioinformatics/btq461 20709691

[ref36] Evans P. N. Boyd J. A. Leu A. O. Woodcroft B. J. Parks D. H. Hugenholtz P. . (2019). An evolving view of methane metabolism in the Archaea. Nat. Rev. Microbiol. 17, 219–232. doi: 10.1038/s41579-018-0136-7, 30664670

[ref37] Farhadian Babadi M. Mehrabi B. Tassi F. Cabassi J. Pecchioni E. Shakeri A. . (2021). Geochemistry of fluids discharged from mud volcanoes in SE Caspian Sea (Gorgan plain, Iran). Int. Geol. Rev. 63, 437–452. doi: 10.1080/00206814.2020.1716400

[ref38] Frank Y. A. Kadnikov V. V. Lukina A. P. Banks D. Beletsky A. V. Mardanov A. V. . (2016). Characterization and genome analysis of the first facultatively alkaliphilic Thermodesulfovibrio isolated from the deep terrestrial subsurface. Front. Microbiol. 7:2000. doi: 10.3389/fmicb.2016.02000, 28066337 PMC5165239

[ref39] Freches A. Fradinho J. C. (2024). The biotechnological potential of the Chloroflexota phylum. Appl. Environ. Microbiol. 90:e01756–23. doi: 10.1128/aem.01756-23, 38709098 PMC11218635

[ref40] Gautam S. B. (2018). “Anaerobic methane oxidizing microbial Community in a Coastal Marine Sediment: anaerobic Methanotrophy dominated by ANME-3,” in Performance Assessment and Enrichment of Anaerobic Methane Oxidizing Microbial Communities from Marine Sediments in Bioreactors, (Boca Raton: CRC Press), 107–144, doi: 10.1201/9780429448027

[ref41] Ghosh W. Dam B. (2009). Biochemistry and molecular biology of lithotrophic sulfur oxidation by taxonomically and ecologically diverse bacteria and archaea. FEMS Microbiol. Rev. 33, 999–1043. doi: 10.1111/j.1574-6976.2009.00187.x, 19645821

[ref42] Guindon S. Dufayard J. F. Lefort V. Anisimova M. Hordijk W. Gascuel O. (2010). New algorithms and methods to estimate maximum-likelihood phylogenies: assessing the performance of PhyML 3.0. Syst. Biol. 59, 307–321. doi: 10.1093/sysbio/syq010, 20525638

[ref43] Hamm J. N. Dombrowski N. Valentin-Alvarado L. E. Greening C. Williams T. A. Spang A. (2026). New lineages provide insights into the convergent evolution of extreme salt adaptation within symbiotic Archaea. Mol. Biol. Evol. 43:msag091. doi: 10.1093/molbev/msag091, 42139541 PMC13195033

[ref44] Haroon M. F. Hu S. Shi Y. Imelfort M. Keller J. Hugenholtz P. . (2013). Anaerobic oxidation of methane coupled to nitrate reduction in a novel archaeal lineage. Nature 500, 567–570. doi: 10.1038/nature12375, 23892779

[ref45] Huang H. Li N. Wang Q. Chen D. (2015). Geochemical features and origins of pore fluids and sediments of the mud volcanoes in southern margin of the Junggar Basin, Xinjiang, northwestern China. Geotecton. Metallog. 39, 325–333. doi: 10.16539/j.ddgzyckx.2015.02.012

[ref46] Hyatt D. Chen G. L. LoCascio P. F. Land M. L. Larimer F. W. Hauser L. J. (2010). Prodigal: prokaryotic gene recognition and translation initiation site identification. BMC Bioinformatics 11:119. doi: 10.1186/1471-2105-11-119, 20211023 PMC2848648

[ref47] Ijiri A. Setoguchi R. Mitsutome Y. Toki T. Murayama M. Hagino K. . (2023). Origins of sediments and fluids in submarine mud volcanoes off Tanegashima Island, northern Ryukyu trench, Japan. Front. Earth Sci. 11:1206810. doi: 10.3389/feart.2023.1206810

[ref48] Judd A. (2005). “Gas emissions from mud volcanoes,” in Mud Volcanoes, Geodynamics and Seismicity Dordrecht: Springer, 147–157. doi: 10.1007/1-4020-3204-8_13

[ref49] Kadnikov V. V. Mardanov A. V. Beletsky A. V. Karnachuk O. V. Ravin N. V. (2020). Microbial life in the deep subsurface aquifer illuminated by metagenomics. Front. Microbiol. 11:572252. doi: 10.3389/fmicb.2020.572252, 33013807 PMC7509429

[ref50] Kalyuzhnaya M. G. Khmelenina V. Eshinimaev B. Sorokin D. Fuse H. Lidstrom M. . (2008). Classification of halo (alkali) philic and halo (alkali) tolerant methanotrophs provisionally assigned to the genera *Methylomicrobium* and *Methylobacter* and emended description of the genus *Methylomicrobium*. Int. J. Syst. Evol. Microbiol. 58, 591–596. doi: 10.1099/ijs.0.65317-0, 18319461

[ref51] Kanai T. Matsuoka R. Beppu H. Nakajima A. Okada Y. Atomi H. . (2011). Distinct physiological roles of the three [NiFe]-hydrogenase orthologs in the hyperthermophilic archaeon *Thermococcus kodakarensis*. J. Bacteriol. 193, 3109–3116. doi: 10.1128/jb.01072-10, 21515783 PMC3133214

[ref52] Kang D. D. Li F. Kirton E. Thomas A. Egan R. An H. . (2019). MetaBAT 2: an adaptive binning algorithm for robust and efficient genome reconstruction from metagenome assemblies. PeerJ 7:e7359. doi: 10.7717/peerj.7359, 31388474 PMC6662567

[ref53] Kappler A. Bryce C. Mansor M. Lueder U. Byrne J. M. Swanner E. D. (2021). An evolving view on biogeochemical cycling of iron. Nat. Rev. Microbiol. 19, 360–374. doi: 10.1038/s41579-020-00502-7, 33526911

[ref54] Kevorkian R. T. Callahan S. Winstead R. Lloyd K. G. (2021). ANME-1 archaea may drive methane accumulation and removal in estuarine sediments. Environ. Microbiol. Rep. 13, 185–194. doi: 10.1111/1758-2229.12926, 33462984

[ref55] Khomyakova M. A. Merkel A. Y. Kopitsyn D. S. Slobodkin A. I. (2022). *Pelovirga terrestris* gen. Nov., sp. nov., anaerobic, alkaliphilic, fumarate-, arsenate-, Fe (III)-and sulfur-reducing bacterium isolated from a terrestrial mud volcano. Syst. Appl. Microbiol. 45:126304. doi: 10.1016/j.syapm.2022.126304, 35144086

[ref56] Khomyakova M. A. Merkel A. Y. Kopitsyn D. S. Slobodkin A. I. (2026). *Desulfurivibrio nitratireducens* sp. nov. and *Desulfurivibrio modestus* sp. nov., two novel alkaliphilic anaerobes isolated from terrestrial mud volcanoes. Syst. Appl. Microbiol. 49:126693. doi: 10.1016/j.syapm.2026.126693, 41643531

[ref57] Khomyakova M. A. Merkel A. Y. Segliuk V. S. Slobodkin A. I. (2023). *Desulfatitalea alkaliphila* sp. nov., an alkalipilic sulfate-and arsenate-reducing bacterium isolated from a terrestrial mud volcano. Extremophiles 27:12. doi: 10.1007/s00792-023-01297-0, 37178152

[ref58] Klasek S. A. Torres M. E. Loher M. Bohrmann G. Pape T. Colwell F. S. (2019). Deep-sourced fluids from a convergent margin host distinct subseafloor microbial communities that change upon mud flow expulsion. Front. Microbiol. 10:1436. doi: 10.3389/fmicb.2019.01436, 31281306 PMC6596357

[ref59] Knittel K. Boetius A. (2009). Anaerobic oxidation of methane: progress with an unknown process. Ann. Rev. Microbiol. 63, 311–334. doi: 10.1146/annurev.micro.61.080706.093130, 19575572

[ref60] Koch T. Dahl C. (2018). A novel bacterial sulfur oxidation pathway provides a new link between the cycles of organic and inorganic sulfur compounds. ISME J. 12, 2479–2491. doi: 10.1038/s41396-018-0209-7, 29930335 PMC6155103

[ref61] Kopf A. J. (2002). Significance of mud volcanism. Rev. Geophys. 40, 2–1. doi: 10.1029/2000RG000093

[ref62] Kraft B. Strous M. Tegetmeyer H. E. (2011). Microbial nitrate respiration–genes, enzymes and environmental distribution. J. Biotechnol. 155, 104–117. doi: 10.1016/j.jbiotec.2010.12.025, 21219945

[ref63] Langmead B. Salzberg S. L. (2012). Fast gapped-read alignment with bowtie 2. Nat. Methods 9, 357–359. doi: 10.1038/nmeth.1923, 22388286 PMC3322381

[ref64] Laso-Pérez R. Wu F. Crémière A. Speth D. R. Magyar J. S. Zhao K. . (2023). Evolutionary diversification of methanotrophic ANME-1 archaea and their expansive virome. Nat. Microbiol. 8, 231–245. doi: 10.1038/s41564-022-01297-4, 36658397 PMC9894754

[ref65] Lavrushin V. Y. Aydarkozhina A. S. Sokol E. V. Chelnokov G. A. Petrov O. L. (2021). Mud volcanic fluids of the Kerch–Taman region: geochemical reconstructions and regional trends. Communication 1: geochemical features and genesis of mud-volcanic waters. Lithol. Miner. Resour. 56, 461–486. doi: 10.1134/S0024490221060043

[ref66] Lavrushin V. Y. Guliev I. S. Kikvadze O. E. Aliev A. A. Pokrovsky B. G. Polyak B. G. (2015). Waters from mud volcanoes of Azerbaijan: isotopic-geochemical properties and generation environments. Lithol. Miner. Resour. 50, 1–25. doi: 10.1134/S0024490215010034

[ref67] Lavrushin V. Y. Kopf A. Deyhle A. Stepanets M. I. (2003). Formation of mud-volcanic fluids in Taman (Russia) and Kakhetia (Georgia): evidence from boron isotopes. Lithol. Miner. Resour. 38, 120–153. doi: 10.1023/A:1023452025440

[ref68] Lazar C. S. Schmidt F. Elvert M. Heuer V. B. Hinrichs K. U. Teske A. P. (2022). Microbial diversity gradients in the geothermal mud volcano underlying the hypersaline Urania Basin. Front. Microbiol. 13:1043414. doi: 10.3389/fmicb.2022.1043414, 36620052 PMC9812581

[ref69] Leu A. O. Cai C. McIlroy S. J. Southam G. Orphan V. J. Yuan Z. . (2020). Anaerobic methane oxidation coupled to manganese reduction by members of the *Methanoperedenaceae*. ISME J. 14, 1030–1041. doi: 10.1038/s41396-020-0590-x, 31988473 PMC7082337

[ref70] Liang Q. Deng L. Xie R. Liu X. Xiao X. Hou J. . (2026). Rapid de novo assembly of animal-microbe biofilter to mitigate seabed methane leakage. Natl. Sci. Rev. 13:nwag266. doi: 10.1093/nsr/nwag266, 42369337 PMC13296563

[ref71] Liao Y. Smyth G. K. Shi W. (2014). featureCounts: an efficient general purpose program for assigning sequence reads to genomic features. Bioinformatics 30, 923–930. doi: 10.1093/bioinformatics/btt656, 24227677

[ref72] Lin Y. T. Tu T. H. Wei C. L. Rumble D. Lin L. H. Wang P. L. (2018). Steep redox gradient and biogeochemical cycling driven by deeply sourced fluids and gases in a terrestrial mud volcano. FEMS Microbiol. Ecol. 94:fiy171. doi: 10.1093/femsec/fiy171, 30165492

[ref73] Liu R. Wei X. Song W. Wang L. Cao J. Wu J. . (2022). Novel Chloroflexi genomes from the deepest ocean reveal metabolic strategies for the adaptation to deep-sea habitats. Microbiome 10:75. doi: 10.1186/s40168-022-01263-6, 35538590 PMC9088039

[ref74] Lösekann T. Knittel K. Nadalig T. Fuchs B. Niemann H. Boetius A. . (2007). Diversity and abundance of aerobic and anaerobic methane oxidizers at the Haakon Mosby mud volcano, Barents Sea. Appl. Environ. Microbiol. 73, 3348–3362. doi: 10.1128/AEM.00016-07, 17369343 PMC1907091

[ref75] Magnabosco C. Lin L. H. Dong H. Bomberg M. Ghiorse W. Stan-Lotter H. . (2018). The biomass and biodiversity of the continental subsurface. Nat. Geosci. 11, 707–717. doi: 10.1038/s41561-018-0221-6

[ref76] Mardanov A. V. Kadnikov V. V. Beletsky A. V. Ravin N. V. (2020). Sulfur and methane-oxidizing microbial community in a terrestrial mud volcano revealed by metagenomics. Microorganisms 8:1333. doi: 10.3390/microorganisms8091333, 32878336 PMC7565565

[ref77] Martin M. (2011). Cutadapt removes adapter sequences from high-throughput sequencing reads. EMBnet. J. 17, 10–12. doi: 10.14806/ej.17.1.200

[ref78] Martinez R. J. Mills H. J. Story S. Sobecky P. A. (2006). Prokaryotic diversity and metabolically active microbial populations in sediments from an active mud volcano in the Gulf of Mexico. Environ. Microbiol. 8, 1783–1796. doi: 10.1111/j.1462-2920.2006.01063.x, 16958759

[ref79] Mayr M. J. Parra S. A. Connon S. A. Narayanan A. K. Murali R. Crémière A. . (2025). Distinct microbial communities within and on seep carbonates support long-term anaerobic oxidation of methane and divergent pMMO diversity. ISME J. 19:wraf153. doi: 10.1093/ismejo/wraf153, 40910361 PMC12422003

[ref80] Mazzini A. Etiope G. (2017). Mud volcanism: an updated review. Earth-Sci. Rev. 168, 81–112. doi: 10.1016/j.earscirev.2017.03.001

[ref81] Megyes M. Móga J. Strat D. Borsodi A. K. (2021). Bacterial and archaeal taxonomic diversity of mud volcanoes (Beciu, Romania) via metagenomic approach. Geomicrobiol J. 38, 532–539. doi: 10.1080/01490451.2021.1900460

[ref82] Merkel A. Y. Chernyh N. A. Pimenov N. V. Bonch-Osmolovskaya E. A. Slobodkin A. I. (2021). Diversity and metabolic potential of the terrestrial mud volcano microbial community with a high abundance of archaea mediating the anaerobic oxidation of methane. Life 11:953. doi: 10.3390/life11090953, 34575103 PMC8470020

[ref83] Meyer B. Kuever J. (2007). Molecular analysis of the diversity of sulfate-reducing and sulfur-oxidizing prokaryotes in the environment, using aprA as functional marker gene. Appl. Environ. Microbiol. 73, 7664–7679. doi: 10.1128/AEM.01272-07, 17921272 PMC2168068

[ref84] Miyake N. Ishimaru R. Komatsu G. Matsui T. (2023). Characterization of archaeal and bacterial communities thriving in methane-seeping on-land mud volcanoes, Niigata. Japan. Int. Microbiol. 26, 191–204. doi: 10.1007/s10123-022-00288-z, 36329310

[ref85] Moore M. T. Vinson D. S. Whyte C. J. Eymold W. K. Walsh T. B. Darrah T. H. (2018). Differentiating between biogenic and thermogenic sources of natural gas in coalbed methane reservoirs from the Illinois Basin using noble gas and hydrocarbon geochemistry. Geol. Soc. Lond., Spec. Publ. 468, 151–188. doi: 10.1144/SP468.8

[ref86] Muhlbauer M. E. Gamiz-Hernandez A. P. Kaila V. R. (2021). Functional dynamics of an ancient membrane-bound hydrogenase. J. Am. Chem. Soc. 143, 20873–20883. doi: 10.1021/jacs.1c09356, 34846879 PMC8679088

[ref87] Murray A. E. Freudenstein J. Gribaldo S. Hatzenpichler R. Hugenholtz P. Kämpfer P. . (2020). Roadmap for naming uncultivated Archaea and Bacteria. Nat. Microbiol. 5, 987–994. doi: 10.1038/s41564-020-0733-x, 32514073 PMC7381421

[ref88] Nagarajan V. Tsai H. C. Chen J. S. Koner S. Kumar R. S. Chao H. C. . (2023). Systematic assessment of mineral distribution and diversity of microbial communities and its interactions in the Taiwan subduction zone of mud volcanoes. Environ. Res. 216:114536. doi: 10.1016/j.envres.2022.114536, 36228688

[ref89] Niemann H. (2020). “Mud volcano biogeochemistry,” in Hydrocarbons, Oils and Lipids: Diversity, Origin, Chemistry and Fate, (Cham: Springer International Publishing), 769–780.

[ref90] Niemann H. Lösekann T. De Beer D. Elvert M. Nadalig T. Knittel K. . (2006). Novel microbial communities of the Haakon Mosby mud volcano and their role as a methane sink. Nature 443, 854–858. doi: 10.1038/nature05227, 17051217

[ref91] Nikitenko O. A. Ershov V. V. (2021). Geochemical patterns of mud volcanic waters: reviewed worldwide data. Geochem. Int. 59, 922–937. doi: 10.1134/S0016702921090044

[ref92] Nurk S. Meleshko D. Korobeynikov A. Pevzner P. A. (2017). metaSPAdes: a new versatile metagenomic assembler. Genome Res. 27, 824–834. doi: 10.1101/gr.213959.116, 28298430 PMC5411777

[ref93] Ollivier B. Fardeau M. L. Cayol J. L. Magot M. Patel B. K. Prensier G. . (1998). *Methanocalculus halotolerans* gen. Nov., sp. nov., isolated from an oil-producing well. Int. J. Syst. Evol. Microbiol. 48, 821–828. doi: 10.1099/00207713-48-3-821, 9734036

[ref94] Orcutt B. N. LaRowe D. E. Biddle J. F. Colwell F. S. Glazer B. T. Reese B. K. . (2013). Microbial activity in the marine deep biosphere: progress and prospects. Front. Microbiol. 4:189. doi: 10.3389/fmicb.2013.00189, 23874326 PMC3708129

[ref95] Orphan V. J. House C. H. Hinrichs K. U. McKeegan K. D. DeLong E. F. (2002). Multiple archaeal groups mediate methane oxidation in anoxic cold seep sediments. Proc. Natl. Acad. Sci. USA 99, 7663–7668. doi: 10.1073/pnas.072210299, 12032340 PMC124316

[ref96] Ouboter H. T. Mesman R. Sleutels T. Postma J. Wissink M. Jetten M. S. . (2024). Mechanisms of extracellular electron transfer in anaerobic methanotrophic archaea. Nat. Commun. 15:1477. doi: 10.1038/s41467-024-45758-2, 38368447 PMC10874420

[ref97] Palù M. Basile A. Zampieri G. Treu L. Rossi A. Morlino M. S. . (2022). KEMET–A python tool for KEGG module evaluation and microbial genome annotation expansion. Comput. Struct. Biotechnol. J. 20, 1481–1486. doi: 10.1016/j.csbj.2022.03.015, 35422973 PMC8976094

[ref98] Pan S. Zhao X. M. Coelho L. P. (2023). SemiBin2: self-supervised contrastive learning leads to better MAGs for short-and long-read sequencing. Bioinformatics 39, i21–i29. doi: 10.1093/bioinformatics/btad209, 37387171 PMC10311329

[ref99] Parks D. H. Chuvochina M. Waite D. W. Rinke C. Skarshewski A. Chaumeil P. A. . (2018). A standardized bacterial taxonomy based on genome phylogeny substantially revises the tree of life. Nat. Biotechnol. 36, 996–1004. doi: 10.1038/nbt.4229, 30148503

[ref100] Petriglieri F. Kondrotaite Z. Singleton C. Nierychlo M. Dueholm M. K. Nielsen P. H. (2023). A comprehensive overview of the *Chloroflexota* community in wastewater treatment plants worldwide. mSystems 8:e00667–23. doi: 10.1128/msystems.00667-23, 37992299 PMC10746286

[ref101] Pisa K. Y. Huber H. Thomm M. Müller V. (2007). A sodium ion-dependent A1AO ATP synthase from the hyperthermophilic archaeon *Pyrococcus furiosus*. FEBS J. 274, 3928–3938. doi: 10.1111/j.1742-4658.2007.05925.x, 17614964

[ref102] Pott A. S. Dahl C. (1998). Sirohaem sulfite reductase and other proteins encoded by genes at the dsr locus of *Chromatium vinosum* are involved in the oxidation of intracellular sulfur. Microbiology 144, 1881–1894. doi: 10.1099/00221287-144-7-1881, 9695921

[ref103] Rajendran S. K. Koner S. Hussain B. Tsai H. C. Hseu Z. Y. Chen J. S. . (2025). Mud volcanoes and microbial communities: unraveling the mysteries of formation, features, and occurrence. J. Asian Earth Sci. 290:106635. doi: 10.1016/j.jseaes.2025.106635

[ref104] Ren G. Ma A. Zhang Y. Deng Y. Zheng G. Zhuang X. . (2018). Electron acceptors for anaerobic oxidation of methane drive microbial community structure and diversity in mud volcanoes. Environ. Microbiol. 20, 2370–2385. doi: 10.1111/1462-2920.14128, 29624877

[ref105] Robinson M. D. McCarthy D. J. Smyth G. K. (2010). edgeR: a Bioconductor package for differential expression analysis of digital gene expression data. Bioinformatics 26, 139–140. doi: 10.1093/bioinformatics/btp616, 19910308 PMC2796818

[ref106] Rodriguez-R L. M. Konstantinidis K. T. (2016). The enveomics collection: a toolbox for specialized analyses of microbial genomes and metagenomes (no. e1900v1). PeerJ Preprints 4:e1900v1. doi: 10.7287/peerj.preprints.1900v1

[ref107] Rognes T. Flouri T. Nichols B. Quince C. Mahé F. (2016). VSEARCH: a versatile open source tool for metagenomics. PeerJ 4:e2584. doi: 10.7717/peerj.2584, 27781170 PMC5075697

[ref108] Ruff E. S. (2020). “Microbial communities and metabolisms at hydrocarbon seeps,” in Marine hydrocarbon Seeps: Microbiology and Biogeochemistry of a Global marine Habitat, (Cham: Springer International Publishing), 1–19.

[ref109] Ruff S. E. De Angelis I. H. Mullis M. Payet J. P. Magnabosco C. Lloyd K. G. . (2024). A global comparison of surface and subsurface microbiomes reveals large-scale biodiversity gradients, and a marine-terrestrial divide. Sci. Adv. 10:eadq0645. doi: 10.1126/sciadv.adq0645, 39693444 PMC11654699

[ref110] Ruiz-Blas F. Bartholomäus A. Yang S. Wagner D. Henny C. Russell J. M. . (2024). Metabolic features that select for Bathyarchaeia in modern ferruginous lacustrine subsurface sediments. ISME Commun. 4:ycae112. doi: 10.1093/ismeco/ycae112, 39660009 PMC11631310

[ref111] Sapra R. Bagramyan K. Adams M. W. (2003). A simple energy-conserving system: proton reduction coupled to proton translocation. Proc. Natl. Acad. Sci. USA 100, 7545–7550. doi: 10.1073/pnas.1331436100, 12792025 PMC164623

[ref112] Sato T. Atomi H. Imanaka T. (2007). Archaeal type III RuBisCOs function in a pathway for AMP metabolism. Science 315, 1003–1006. doi: 10.1126/science.1135999, 17303759

[ref113] Schauberger C. Thamdrup B. Lemonnier C. Trouche B. Poulain J. Wincker P. . (2024). Metagenome-assembled genomes of deep-sea sediments: changes in microbial functional potential lag behind redox transitions. ISME Commun. 4:ycad005. doi: 10.1093/ismeco/ycad005, 38282644 PMC10809760

[ref114] Schloss P. D. Westcott S. L. Ryabin T. Hall J. R. Hartmann M. Hollister E. B. . (2009). Introducing mothur: open-source, platform-independent, community-supported software for describing and comparing microbial communities. Appl. Environ. Microbiol. 75, 7537–7541. doi: 10.1128/AEM.01541-09, 19801464 PMC2786419

[ref115] Schut G. J. Boyd E. S. Peters J. W. Adams M. W. (2013). The modular respiratory complexes involved in hydrogen and sulfur metabolism by heterotrophic hyperthermophilic archaea and their evolutionary implications. FEMS Microbiol. Rev. 37, 182–203. doi: 10.1111/j.1574-6976.2012.00346.x, 22713092

[ref116] Shaffer M. Borton M. A. McGivern B. B. Zayed A. A. La Rosa S. L. Solden L. M. . (2020). DRAM for distilling microbial metabolism to automate the curation of microbiome function. Nucleic Acids Res. 48, 8883–8900. doi: 10.1093/nar/gkaa621, 32766782 PMC7498326

[ref117] Shnyukov E. Yanko-Hombach V. (2020). Mud Volcanoes of the Black Sea region and their Environmental Significance. Cham: Springer Nature.

[ref118] Sieber C. M. Probst A. J. Sharrar A. Thomas B. C. Hess M. Tringe S. G. . (2018). Recovery of genomes from metagenomes via a dereplication, aggregation and scoring strategy. Nat. Microbiol. 3, 836–843. doi: 10.1038/s41564-018-0171-1, 29807988 PMC6786971

[ref119] Slobodkin A. I. Rusanov I. I. Slobodkina G. B. Stroeva A. R. Chernyh N. A. Pimenov N. V. . (2024). Diversity, methane oxidation activity, and metabolic potential of microbial communities in terrestrial mud volcanos of the Taman peninsula. Microorganisms 12:1349. doi: 10.3390/microorganisms12071349, 39065117 PMC11279179

[ref120] Slobodkina G. Merkel A. Ratnikova N. Kuchierskaya A. Slobodkin A. (2023). *Sedimenticola hydrogenitrophicus* sp. nov. a chemolithoautotrophic bacterium isolated from a terrestrial mud volcano, and proposal of *Sedimenticolaceae* fam. Nov. in the order Chromatiales. Syst. Appl. Microbiol. 46:126451. doi: 10.1016/j.syapm.2023.126451, 37562281

[ref121] Slobodkina G. Ratnikova N. Merkel A. Kevbrin V. Kuchierskaya A. Slobodkin A. (2022). Lithoautotrophic lifestyle of the widespread genus *Roseovarius* revealed by physiological and genomic characterization of *Roseovarius autotrophicus* sp. nov. FEMS Microbiol. Ecol. 98:fiac113. doi: 10.1093/femsec/fiac113, 36166357

[ref122] Sokol E. Kokh S. Kozmenko O. Novikova S. Khvorov P. Nigmatulina E. . (2018). Mineralogy and geochemistry of mud volcanic ejecta: a new look at old issues (a case study from the Bulganak field, northern Black Sea). Minerals 8:344. doi: 10.3390/min8080344

[ref123] Sorokin D. Y. Tourova T. P. Mußmann M. Muyzer G. (2008). *Dethiobacter alkaliphilus* gen. Nov. sp. nov., and *Desulfurivibrio alkaliphilus* gen. Nov. sp. nov.: two novel representatives of reductive sulfur cycle from soda lakes. Extremophiles 12, 431–439. doi: 10.1007/s00792-008-0148-8, 18317684

[ref124] Steiner Z. Turchyn A. V. Ziveri P. Shiller A. M. Lam P. J. Paytan A. . (2025). The roles of celestine and barite in modulating strontium and barium water column concentrations in the Northeast Pacific Ocean. Geochim. Cosmochim. Acta 388, 182–194. doi: 10.1016/j.gca.2024.10.003

[ref125] Tatusova T. DiCuccio M. Badretdin A. Chetvernin V. Nawrocki E. P. Zaslavsky L. . (2016). NCBI prokaryotic genome annotation pipeline. Nucleic Acids Res. 44, 6614–6624. doi: 10.1093/nar/gkw569, 27342282 PMC5001611

[ref126] Tavormina P. L. Hatzenpichler R. McGlynn S. Chadwick G. Dawson K. S. Connon S. A. . (2015). *Methyloprofundus sedimenti* gen. Nov., sp. nov., an obligate methanotroph from ocean sediment belonging to the ‘deep sea-1’clade of marine methanotrophs. Int. J. Syst. Evol. Microbiol. 65, 251–259. doi: 10.1099/ijs.0.062927-0, 25342114

[ref127] Thauer R. K. Kaster A. K. Seedorf H. Buckel W. Hedderich R. (2008). Methanogenic archaea: ecologically relevant differences in energy conservation. Nat. Rev. Microbiol. 6, 579–591. doi: 10.1038/nrmicro1931, 18587410

[ref128] Tu T. H. Chen L. L. Chiu Y. P. Lin L. H. Wu L. W. Italiano F. . (2022). The biogeographic pattern of microbial communities inhabiting terrestrial mud volcanoes across the Eurasian continent. Biogeosciences 19, 831–843. doi: 10.5194/bg-19-831-2022

[ref129] Tu T. H. Wu L. W. Lin Y. S. Imachi H. Lin L. H. Wang P. L. (2017). Microbial community composition and functional capacity in a terrestrial ferruginous, sulfate-depleted mud volcano. Front. Microbiol. 8:2137. doi: 10.3389/fmicb.2017.02137, 29163423 PMC5673622

[ref130] Tuininga J. E. Verhees C. H. van der Oost J. Kengen S. W. Stams A. J. de Vos W. M. (1999). Molecular and biochemical characterization of the ADP-dependent phosphofructokinase from the hyperthermophilic archaeon *Pyrococcus furiosus*. J. Biol. Chem. 274, 21023–21028. doi: 10.1074/jbc.274.30.21023, 10409652

[ref131] van der Oost J. Schut G. Kengen S. M. Hagen W. R. Thomm M. de Vos W. M. (1998). The ferredoxin-dependent conversion of glyceraldehyde-3-phosphate in the hyperthermophilic archaeon *Pyrococcus furiosus* represents a novel site of glycolytic regulation. J. Biol. Chem. 273, 28149–28154. doi: 10.1074/jbc.273.43.28149, 9774434

[ref132] Wang P. L. Chiu Y. P. Cheng T. W. Chang Y. H. Tu W. X. Lin L. H. (2014). Spatial variations of community structures and methane cycling across a transect of lei-gong-Hou mud volcanoes in eastern Taiwan. Front. Microbiol. 5:121. doi: 10.3389/fmicb.2014.00121, 24723919 PMC3971192

[ref133] Wang Z. You R. Han H. Liu W. Sun F. Zhu S. (2024). Effective binning of metagenomic contigs using contrastive multi-view representation learning. Nat. Commun. 15:585. doi: 10.1038/s41467-023-44290-z, 38233391 PMC10794208

[ref134] Wegener G. Krukenberg V. Ruff S. E. Kellermann M. Y. Knittel K. (2016). Metabolic capabilities of microorganisms involved in and associated with the anaerobic oxidation of methane. Front. Microbiol. 7:46. doi: 10.3389/fmicb.2016.00046, 26870011 PMC4736303

[ref135] Wiegand S. Sobol M. Schnepp-Pesch L. K. Yan G. Iqbal S. Vollmers J. . (2023). Taxonomic re-classification and expansion of the phylum Chloroflexota based on over 5000 genomes and metagenome-assembled genomes. Microorganisms 11:2612. doi: 10.3390/microorganisms11102612, 37894270 PMC10608941

[ref136] Williams S. A. Riley D. M. Rockwood T. P. Crosby D. A. Call K. D. LeCuyer J. J. . (2025). A dynamic protein interactome drives energy conservation and electron flux in *Thermococcus kodakarensis*. Appl. Environ. Microbiol. 91:e00293–25. doi: 10.1128/aem.00293-25, 40178256 PMC12016516

[ref137] Woods P. H. Speth D. R. Laso-Pérez R. Utter D. R. Ruff S. E. Orphan V. J. (2025). Identification of key steps in the evolution of anaerobic methanotrophy in Candidatus Methanovorans (ANME-3) archaea. Sci. Adv. 11:eadq5232. doi: 10.1126/sciadv.adq5232, 40540566 PMC12180501

[ref138] Wrede C. Brady S. Rockstroh S. Dreier A. Kokoschka S. Heinzelmann S. M. . (2012). Aerobic and anaerobic methane oxidation in terrestrial mud volcanoes in the northern Apennines. Sediment. Geol. 263-264, 210–219. doi: 10.1016/j.sedgeo.2011.06.004, 38826717

[ref139] Wu Y. W. Simmons B. A. Singer S. W. (2016). MaxBin 2.0: an automated binning algorithm to recover genomes from multiple metagenomic datasets. Bioinformatics 32, 605–607. doi: 10.1093/bioinformatics/btv638, 26515820

[ref140] Wurch L. Giannone R. J. Belisle B. S. Swift C. Utturkar S. Hettich R. L. . (2016). Genomics-informed isolation and characterization of a symbiotic Nanoarchaeota system from a terrestrial geothermal environment. Nat. Commun. 7:12115. doi: 10.1038/ncomms12115, 27378076 PMC4935971

[ref141] Yi X. Brandt K. K. Xue S. Peng J. Wang Y. Li M. . (2024). Niche differentiation and biogeography of Bathyarchaeia in paddy soil ecosystems: a case study in eastern China. Environ. Microbiome 19:13. doi: 10.1186/s40793-024-00555-8, 38429752 PMC10908009

[ref142] Yokooji Y. Sato T. Fujiwara S. Imanaka T. Atomi H. (2013). Genetic examination of initial amino acid oxidation and glutamate catabolism in the hyperthermophilic archaeon *Thermococcus kodakarensis*. J. Bacteriol. 195, 1940–1948. doi: 10.1128/jb.01979-12, 23435976 PMC3624576

[ref143] Yu H. Speth D. R. Connon S. A. Goudeau D. Malmstrom R. R. Woyke T. . (2022). Community structure and microbial associations in sediment-free methanotrophic enrichment cultures from a marine methane seep. Appl. Environ. Microbiol. 88:e02109–21. doi: 10.1128/aem.02109-21, 35604226 PMC9195934

[ref144] Yu H. Wu C. H. Schut G. J. Haja D. K. Zhao G. Peters J. W. . (2018). Structure of an ancient respiratory system. Cell 173, 1636–1649.e16. doi: 10.1016/j.cell.2018.03.071, 29754813 PMC6003862

[ref145] Yu H. Xu S. Jangir Y. Wegener G. Orphan V. J. El-Naggar M. Y. (2025). Redox conduction facilitates direct interspecies electron transport in anaerobic methanotrophic consortia. Sci. Adv. 11:eadw4289. doi: 10.1126/sciadv.adw4289, 40845095 PMC12372872

[ref146] Zhao D. Zhang S. Kumar S. Zhou H. Xue Q. Sun W. . (2022). Comparative genomic insights into the evolution of Halobacteria-associated “Candidatus Nanohaloarchaeota”. Msystems 7:e00669–22. doi: 10.1128/msystems.00669-22, 36259734 PMC9765267

[ref147] Zhou Z. Tran P. Q. Cowley E. S. Trembath-Reichert E. Anantharaman K. (2025). Diversity and ecology of microbial sulfur metabolism. Nat. Rev. Microbiol. 23, 122–140. doi: 10.1038/s41579-024-01104-3, 39420098

