# Supplementary material for: Metagenomic insights into microbial communities of terrestrial mud volcanos: functional diversity of subsurface archaea and bacteria
Source: Front Microbiol. 2026 Aug 11;17:1892847. doi: 10.3389/fmicb.2026.1892847 (PMC13503563; doi:10.3389/fmicb.2026.1892847)
Supplement: Supplementary file 1 [file Data_Sheet_1.PDF]

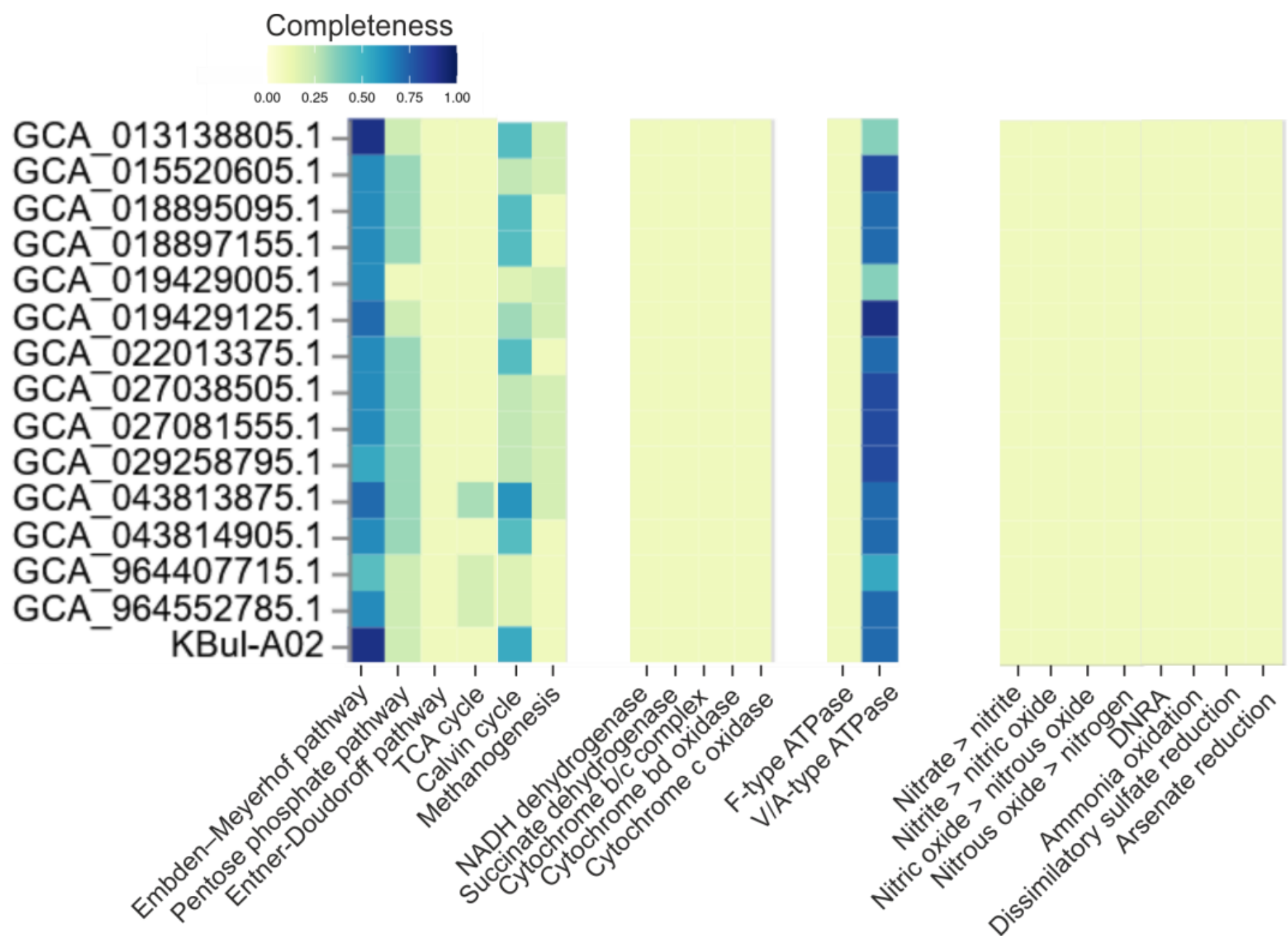

Figure S1. Comparative analysis of the main metabolic pathways along the candidate phylum EX4484-52 performed using the DRAM tool. The completeness of the metabolic pathways and the completion of the subunits of the respiratory complexes are shown using color coding. DNRA, ammonia-forming cytochrome c nitrite reductase.
